# The immunophenotypic landscape of newly diagnosed acute myeloid leukemia: Associations with genotype and outcomes

**DOI:** 10.1016/j.isci.2026.117762

**Published:** 2026-10-12

**Authors:** Tim R. Mocking, Tom Reuvekamp, Lukas H. Haaksma, Eirini Papavasileiou, Angèle Kelder, Willemijn J. Scholten, Dimitri A. Breems, Thomas Fischer, Bjørn T. Gjertsen, Laimonas Griškevičius, Gunnar Juliusson, Johan A. Maertens, Markus G. Manz, Thomas Pabst, Jakob R. Passweg, Kimmo Porkka, Jeroen J.W.M. Janssen, Patrycja Gradowska, Bob Löwenberg, Gert J. Ossenkoppele, Arjan A. van de Loosdrecht, David C. de Leeuw, Jacqueline Cloos, Peter J.M. Valk, Costa Bachas

**Affiliations:** 1 Amsterdam UMC, Amsterdam, the Netherlands; 2 Cancer Center Amsterdam, Amsterdam, the Netherlands; 3 Ziekenhuis aan de Stroom, Antwerpen, Belgium; 4 Otto von Guericke University Hospital Magdeburg, Magdeburg, Germany; 5 Haukeland University Hospital, Bergen, Norway; 6 Vilnius University Hospital Santaros Klinikos and Vilnius University, Vilnius, Lithuania; 7 Skanes University Hospital, Lund, Sweden; 8 University Hospital Gasthuisberg, Leuven, Belgium; 9 University Hospital, Zurich, Switzerland; 10 Swiss Group for Clinical Cancer Research, Bern, Switzerland; 11 University Hospital Inselspital, Bern, Switzerland; 12 University Hospital, Basel, Switzerland; 13 Helsinki University Hospital Cancer Center, Helsinki, Finland; 14 Radboud University Medical Center, Nijmegen, the Netherlands; 15 Erasmus Medical Center Cancer Institute, Rotterdam, the Netherlands; 16 HOVON Foundation, Rotterdam, the Netherlands

**Keywords:** acute myeloid leukemia, immunophenotyping, genomics, flow cytometry, unsupervised clustering, patient stratification, machine learning

## Abstract

Despite widespread immunophenotyping in acute myeloid leukemia (AML), its immunophenotypic heterogeneity remains incompletely understood. Here, we applied state-of-the-art computational modeling to >50 million single cells from diagnostic bone marrow aspirates of 502 AML patients. Jointly modeling leukemic and non-malignant cells, we identified seven patient clusters with distinct immunophenotypic signatures. These clusters showed strong concordance with recurrent mutations, including *NPM1*, *IDH1/IDH2*, *CEBPA*, and t(8;21)(q22;q22); *RUNX1-RUNX1T1* fusions, and enabled accurate prediction of genomic subgroups. Although these immunophenotypic signatures were not associated with therapy responses, loss of HLA-DR expression on leukemic blasts at diagnosis was independently associated with inferior outcomes in patients with chromatin-spliceosome mutations. In addition, we identified an immunophenotypic patient cluster comprising ∼11% of patients characterized by significant bone marrow lymphocyte enrichment where outcomes diverged depending on genetic risk. Together, these findings provide a comprehensive single-cell characterization of AML bone marrow and reveal clinically relevant genotype-phenotype relationships.

## Highlights

• Bone marrow immunophenotyping reveals seven AML patient clusters at diagnosis
• Immunophenotypic signatures correlate with recurrent genetic AML lesions
• Loss of HLA-DR expression associates with inferior outcomes in chromatin-spliceosome AML
• Prognostic relevance of bone marrow lymphocyte enrichment, depending on genetic risk

## Graphical abstract

**Figure:**
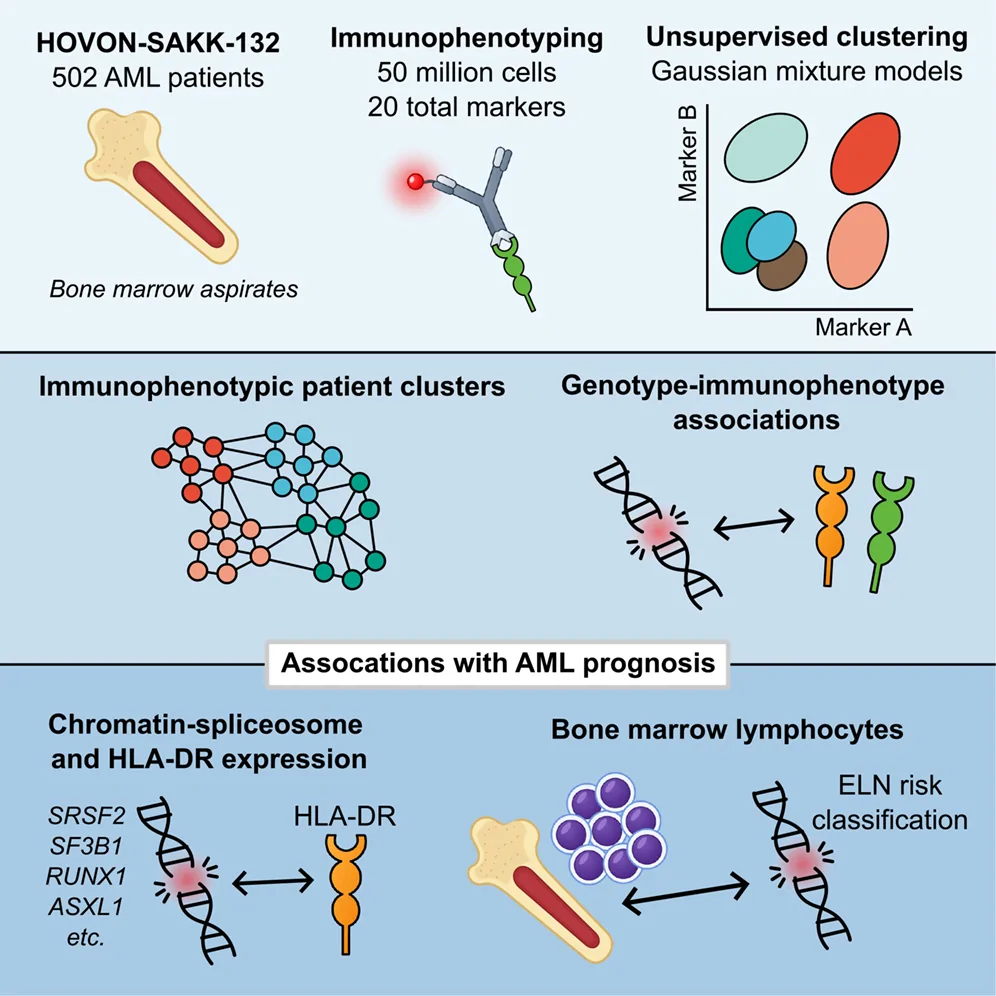

## Teaser

Health sciences

## Introduction

Acute myeloid leukemia (AML) is a heterogeneous myeloid neoplasm at the (epi)genetic and phenotypic level. Within individual cases, clonal heterogeneity provides the foundation for the emergence of therapy resistance, resulting in high relapse rates. The variety of driver events and their downstream pathways also limits the applicability of targeted therapies to small groups of patients. Although different omics technologies have provided significant insights into the heterogeneity of AML across (epi)genetic, transcriptomic, metabolic, and proteomic modalities,^1^^,^^2^ an urgent need remains to identify the appropriate AML patient characteristics for personalized medicine.

While the European leukemia net (ELN) risk stratification of AML only considers genetic lesions,^3^ recent studies have shown that immunophenotypic features of leukemic blasts can be predictive for responses to targeted therapies such as venetoclax^4^^,^^5^ as well as intensive chemotherapy.^6^^,^^7^ Additionally, altered immune responses in the bone marrow (BM) microenvironment have been linked to inferior responses and outcomes for intensive chemotherapy.^8^ Beyond genetic features for risk stratification, leukemic blasts are known to express leukemia-associated immunophenotypes (LAIPs), cell-surface antigen expression patterns that are rare or absent on healthy progenitors. In AML, LAIPs such as overexpression of myeloid markers (e.g., CD123,^9^ CD33^10^), and aberrantly expressed proteins (e.g., CD7,^11^ CD19,^12^ CLEC12A,^13^ and TIM3^14^) can directly function as targets for therapy, even on leukemia stem cells (LSCs) that can initiate and maintain the disease.^15^ However, the large number of LAIPs already identified in AML suggests that the profound heterogeneity at the genetic level is also found at the immunophenotypic level. Therefore, risk stratification for novel therapies and effective targeted therapies directed against cell-surface characteristics necessitates improved patient characterization to identify subgroups where these therapies are most likely to succeed.

Several studies have identified associations between driver mutations and specific LAIPs.^16^^,^^17^^,^^18^ However, despite broad implementation of immunophenotyping in clinical diagnostics and measurable residual disease (MRD) assessment,^3^^,^^19^^,^^20^ predefined manual flow cytometry analysis (gating) and insufficient assay standardization have limited these studies to smaller cohorts with few markers. Consequently, our understanding of the inter- and intra-sample immunophenotypic heterogeneity in AML and its relationship with other biological and clinical modalities remains limited. To fill this gap, we applied extensive unsupervised modeling to BM aspirates from 502 newly diagnosed AML patients, to enable an in-depth characterization of the immunophenotypic landscape of AML.

## Results

### Dissecting the immunophenotypic landscape of AML

To characterize the immunophenotypic landscape of AML, we performed large-scale clustering of BM cells from 502 newly diagnosed AML patients and 20 normal bone marrow (NBM) controls (Figure 1A). Cells were stained using a five-tube flow cytometry assay^21^ (Table S1) that allowed for the detection of hematopoietic stem cell and progenitor cell (HSPC), myeloid lineage, and LAIP markers. In contrast to popular cytometry clustering algorithms (e.g., FlowSOM,^22^ PhenoGraph^23^), we used the computationally efficient multi-sample Gaussian mixture model (MSGMM) algorithm^24^ to avoid balanced downsampling and retain sample-level information during clustering. We previously demonstrated that MSGMM improved the detection of cell populations with highly variable inter-sample abundance that are intrinsic to AML compared to contemporary methods.^24^ The computational efficiency of MSGMM also facilitated modeling more than 50 million single cells per tube (Table S2). In each of the five tubes, we categorized cells into 100 clusters with an over-clustering strategy to ensure the detection of rare cell populations.^25^^,^^26^ Because different immunophenotypes such as monocytic AML fall outside the canonical CD45-dim blast compartment, we included all clusters within the WBC compartment (*n* = 475 clusters; Figure S1) to capture a comprehensive immunophenotypic overview.

**Figure 1:**
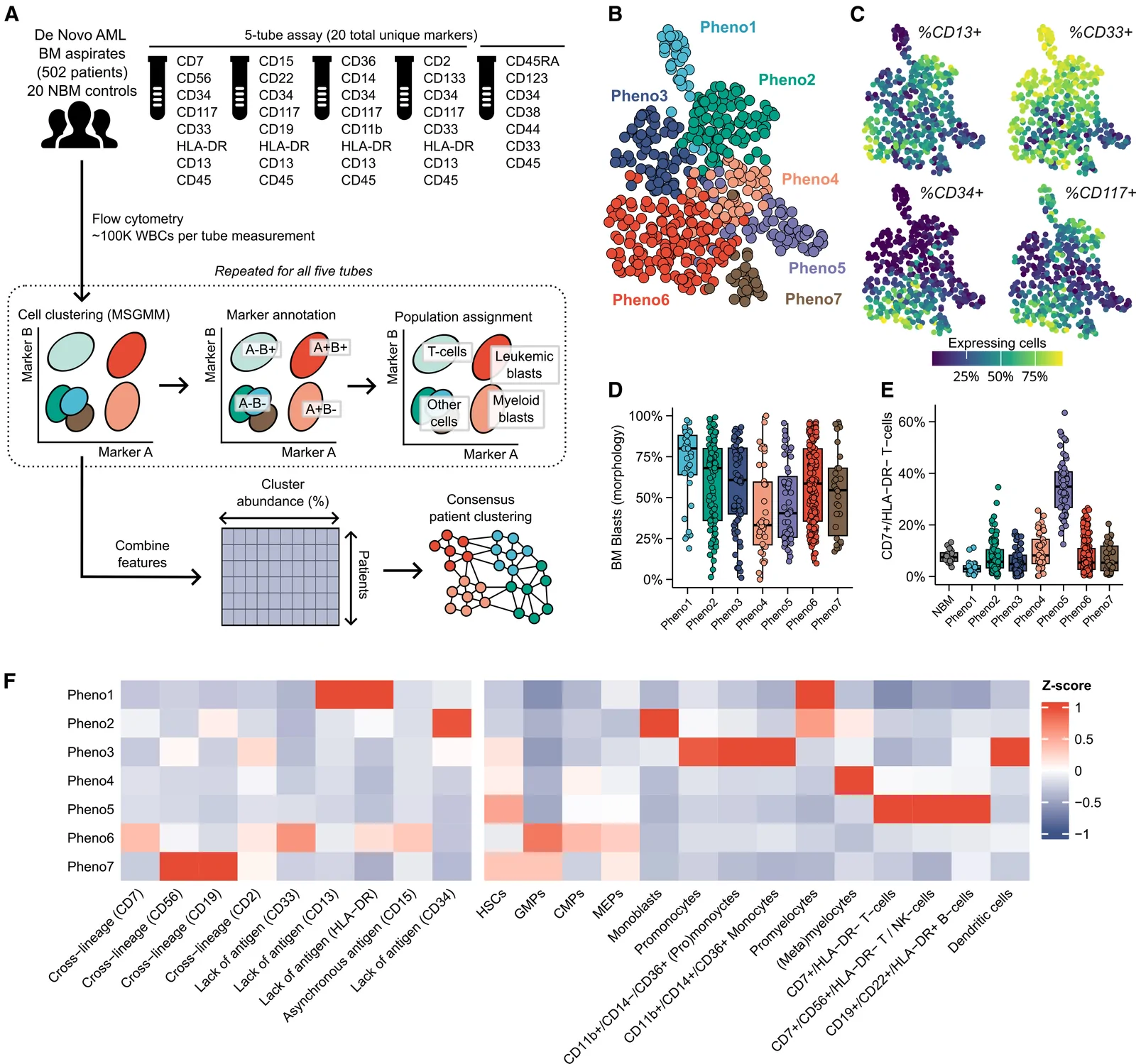
AML patients can be stratified by seven distinct immunophenotypic patient clusters (A) Schematical overview of consensus clustering approach. (B) UMAP of cluster proportions colored by immunophenotypic patient clusters identified by consensus clustering. (C) WBC percentages of clusters expressing key myeloid and primitive markers (CD13, CD33, CD34, and CD117) in each patient. If markers were present in multiple tubes, the mean proportion across tubes was used. (D) Morphological blast percentages for every patient cluster. (E) Abundance of CD7^+^ lymphocytes (% of WBCs) in every patient cluster and NBM controls. (F) Heatmap of immunophenotypic characteristics (left: LAIPs; right: healthy subsets) of each patient cluster. Data are represented as median ± IQR.

Next, we leveraged the statistical nature of the model to link WBC clusters to known cell populations (Figures 1A and S2). This enabled a rough characterization of HSPC, maturing monocyte and neutrophil, and immune subsets (Table S3). Additionally, by matching cluster annotations to established LAIPs (Table S4), we were able to identify 41 clusters that would be characterized as leukemic and tracked longitudinally for MRD assessment in clinical practice. Besides manual annotation, LAIPs were also determined in data-driven fashion by quantifying the background abundance (defined as mean +3 SD) in 20 NBM samples (Figure S3). This showed that the majority (27/41; 66%) of manually annotated LAIP-matched clusters had low abundance (<0.1% of WBCs) in NBM. The data-driven LAIP assessment also identified 56 cell clusters with low background in NBM that were not matched to known LAIPs. However, the subtle and high-dimensional nature of these aberrancies complicated further conventional investigation based on low-dimensional characteristics. Therefore, we focused on the 41 cell clusters that could be unequivocally linked to established LAIPs in further analysis.

### AML patients can be stratified into seven distinct immunophenotypic patient clusters

To gain an understanding of shared immunophenotypic characteristics, AML patients were grouped using consensus clustering based on relative cell abundances of all WBC clusters (Figures 1B and S4). We identified seven patient clusters (Pheno1-7) that differ primarily in the abundance of cell clusters expressing myeloid markers, distinguishing between predominantly immature (CD34^+^; Pheno6,7) and mature (CD34^−^, Pheno1-3) patient clusters (Figure 1C). Immunophenotypic classification was partially concordant with the French-American British (FAB) morphological classification (Figure S5), with 75% of patients with the primarily monocytic immunophenotype (CD34^−^/CD117^−^; Figure 1C) typed as (myelo)monocytic FAB (M4/M5), while the more immature clusters (Pheno6, Pheno7) were predominantly FAB M0-M2 (acute myeloblastic).

As patient clusters were based on the relative proportions of all their WBC clusters, they also reflected differences in morphological blast counts. Patients in Pheno4 and Pheno5 had relatively few blasts (median 27% and 30%, respectively) and proportionally fewer clusters expressing myeloid markers (Figure 1C), while patients with Pheno1 were diagnosed with a median blast count of 85%. Among the low blast clusters, Pheno5 was enriched for lymphocytes when compared to other patient clusters and NBM (Figure 1E). In addition, each patient cluster featured a unique immunophenotypic signature (Figure 1F, unaggregated LAIPs; Figure S6) that was explored further in more detail.

### Co-mutational patterns and chromosomal abnormalities are associated with distinct cell-surface characteristics

To evaluate the associations between the immunophenotypic patient clusters and genotypes, we classified all patients with complete genotyping into genomic subgroups according to a previous classification by Papaemmanuil et al.^27^ Patient assignments (Table S5), co-mutational patterns (Figure S7), and relative differences in overall survival (OS) (Figure S8) in this cohort closely resembled previously published characteristics.

The immunophenotypic and genomic classification showed several instances of clear concordance. Pheno1-3 and Pheno7 comprised almost exclusively *NPM1* and t(8;21) patients, respectively (Figure 2A). Within the *NPM1* patients, we found more granular genotype-phenotype associations, as Pheno1 (*n* = 33) consisted almost exclusively of *NPM1* (32/33) and *IDH1*/*IDH2*^R140^ mutated (30/33) patients, but never *IDH2*^R172^ (Figure S9). Other genomic subgroups showed greater inter-patient similarity but clustered together within single immunophenotypic clusters (Figure 2B). For example, patients with inv(16), *CEBPA*^biallelic^ and *IDH2*^R172^ clustered almost exclusively within Pheno6 (>79%). Collectively, our results demonstrate that several known genomic subgroups result in distinct cell-surface characteristics that drive our immunophenotypic classification.

**Figure 2:**
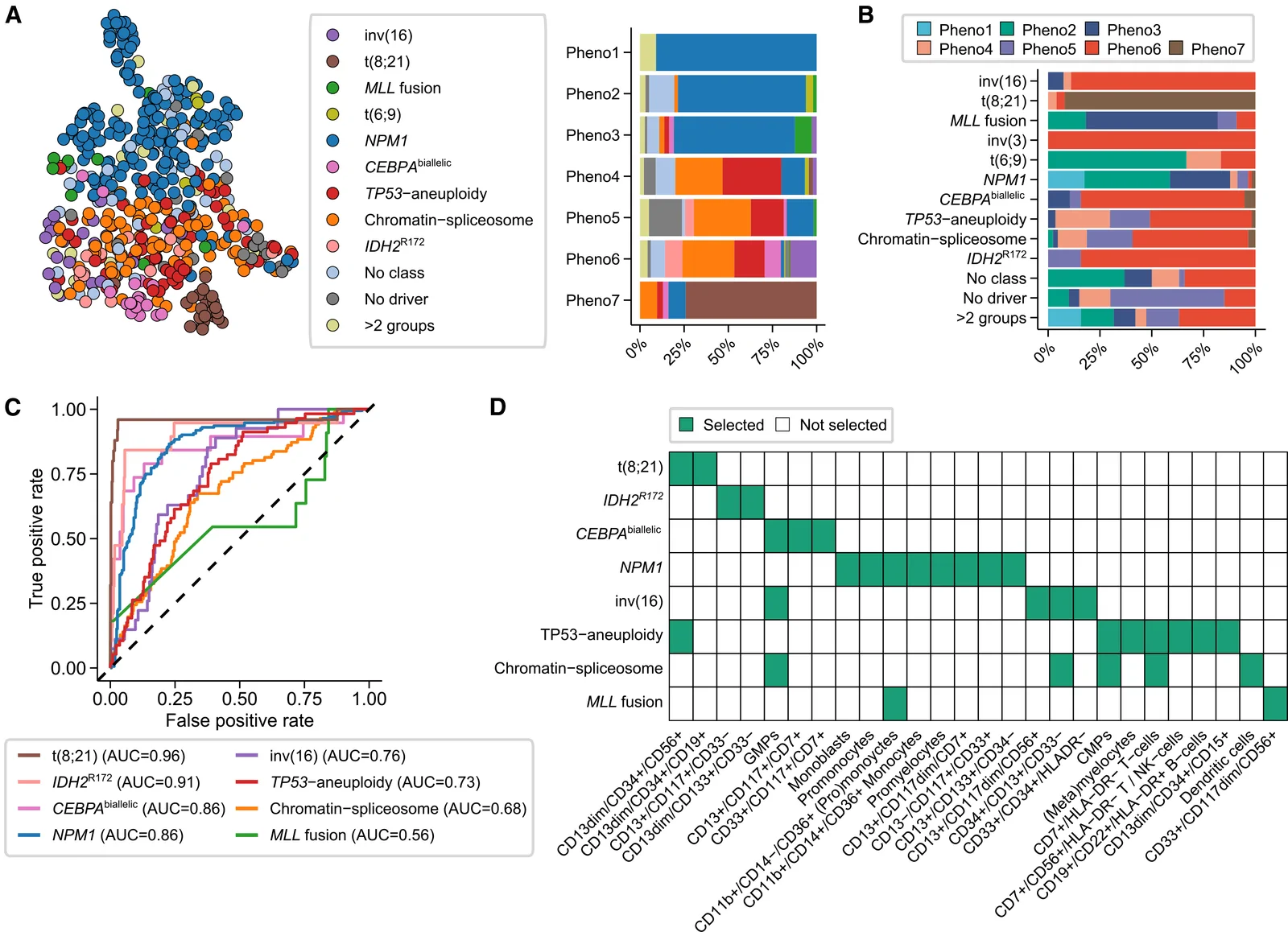
Genotype-immunophenotype associations in AML (A) UMAP of cluster proportions colored by genomic subgroups and relative proportions of each genomic subgroup with immunophenotypic patient clusters. (B) Relative proportions of immunophenotypic patient clusters across genomic subgroups. (C) Receiver under the operating characteristic (ROC) curve for machine learning predictions of genomic subgroups based on immunophenotypic features. (D) Features selected by L1-regularized logistic regression for genomic subgroup prediction. All features have positive associations (enrichment) in the genomic subgroup.

### Genomic subgroups can be predicted from immunophenotypic characteristics

To further quantify genotype-phenotype associations, we trained a classifier to predict genomic subgroups from known cell populations (Tables S3 and S4). This revealed that some groups, such as t(8;21), *IDH2*^R172^, *CEBPA*^biallelic^, and *NPM1*, are highly predictable (ROC-AUC >0.85) based on immunophenotypic features alone (Figure 2C). Subgroups with more genetic heterogeneity, such as Chromatin-spliceosome, *TP53*-aneuploidy, and MLL fusion subgroups, were among the least well predicted (ROC-AUC <0.75; Figure 2C), reflecting their heterogeneity (Figure 2A). Immunophenotypic features selected based on predictive power for genomic subgroup prediction reflected genotype-immunophenotype associations (Figure 2D) and were robust across repeated cross-validation iterations (Table S6). This included known associations such as t(8;21) and cross-lineage expression of CD56 and CD19^16^, *CEBPA*^biallelic^ and cross-lineage expression of CD7,^17^ and *IDH2*^R172^ patients with aberrant loss of CD33.^18^

Although the *NPM1* genetic subgroup could be accurately predicted based on immunophenotypic features (AUC = 0.86), relatively many features (*n* = 8) were required (Figure 2D). Among these were characteristic enrichments for neutrophilic and monocytic populations (Figure 2D). These populations may be malignant in *NPM1* mutated patients but were not distinguishable from healthy cells in our panel due to the absence of mature LAIP markers such as CD4 and CD64. Nevertheless, the model selected three characteristic LAIPs for *NPM1*: cross-lineage expression of CD7 on CD13^+^/CD117dim cells (primarily found in Pheno2), aberrant loss of CD13 on CD117^+^/CD33^+^ cells (primarily found in Pheno1), and CD34 on CD13^+^/CD133^+^ cells (primarily found in Pheno2; Figure S6). Beyond CD34 negativity and aberrant under expression of CD13 for the *NPM1/IDH1/IDH2*^*R140*^ mutational signature in Pheno1, we found that these patients often showed aberrant loss of HLA-DR expression (Figure S6), characteristics that were previously associated with acute promyelocytic leukemia.^28^ While no characteristic LAIPs were selected in the model to predict the primarily CD34^−^/CD117^−^monocytic-like patients in Pheno3 (Figures 2B and 2C), cross-lineage expression of CD56 on CD117^dim^ cells was observed (Figure S6).

Two HSPC-like subsets were also informative for prediction of genomic subgroups: CD45RA^+^/CD123^+^/CD34^+^/CD38^+^ GMPs (inv(16), *CEBPA*^biallelic^ and chromatin-spliceosome) and CD45RA^−^/CD123^+^/CD34^+^/CD38^+^/CD44^+^ CMPs (*TP53*-aneuploidy, chromatin-spliceosome). We hypothesize that these were selected due to the increased abundance of immature blasts associated with these genomic subgroups (Figures 1C and 2A).

### Loss of HLA-DR on leukemic blasts is independently associated with inferior outcomes for patients with chromatin-spliceosome mutations

To evaluate prognostic relevance, we first evaluated associations of the immunophenotypic patient clusters with patient outcomes. Differences in OS were minimal (Figure 3A), especially when compared to genomic classification (Figure S8). Additionally, stratification of the largest genomic subgroup, the primarily CD34-negative patients with (*NPM1*; *n* = 172), into the APL-like phenotype of Pheno1 (CD117^+^/CD13^−^/CD33^+^), Pheno2 (CD117^+^/CD13^+^/CD33^+^), and the monocytic-like Pheno3 (CD117^−^/CD13^+^/CD33^+^) did not show significant differences in OS (Figure 3B). Similarly, we also did not observe survival differences when stratifying the second largest group with chromatin-spliceosome mutations (Figure 3C).

**Figure 3:**
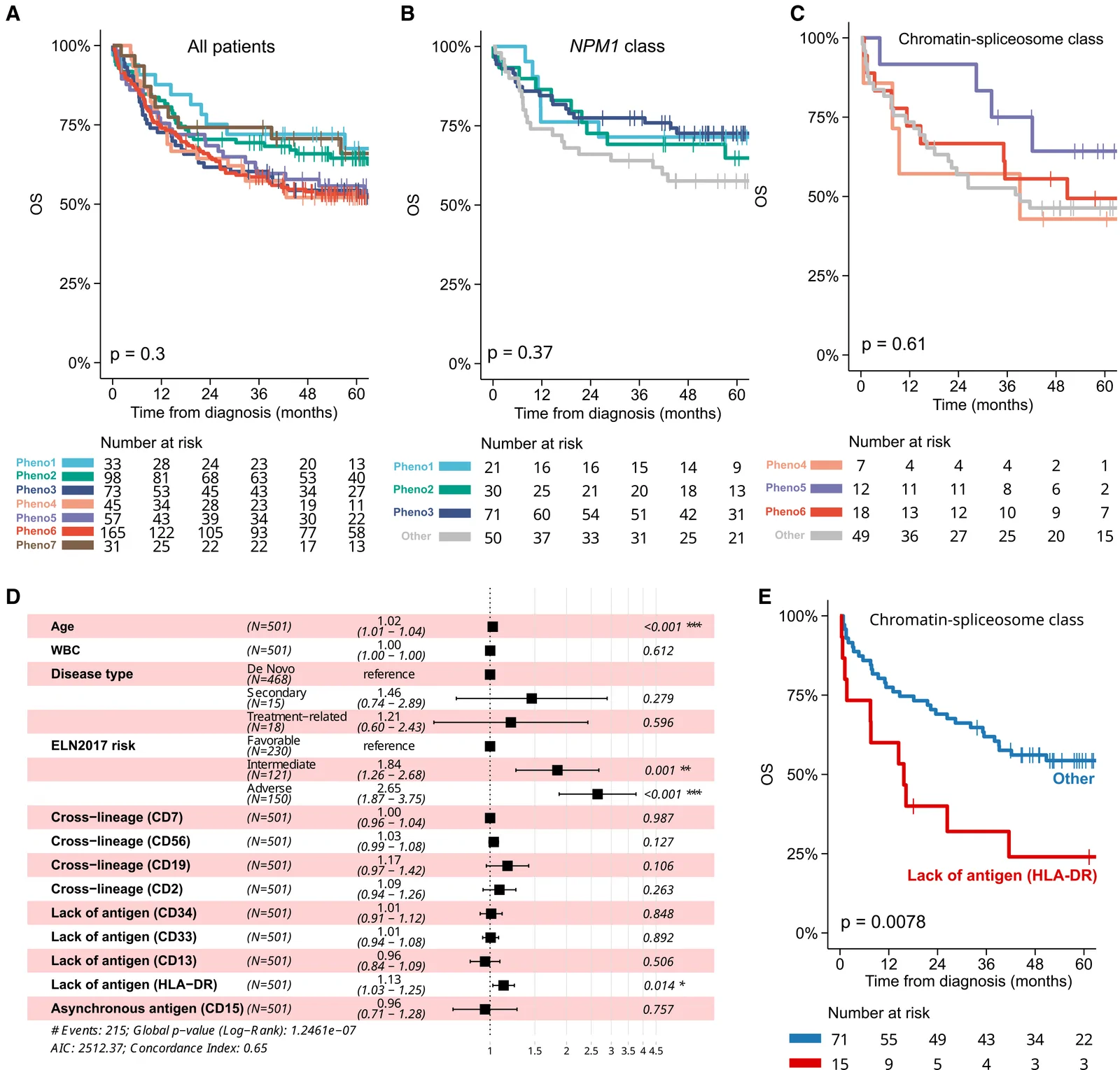
Complementary prognostic value of immunophenotypic characteristics (A–C) Kaplan Meier curves for OS for all patients (A), patients with the *NPM1* genomic class (B) and patients with the chromatin-spliceosome genomic class (C). Patients classified as “other” belong to other immunophenotypic patient clusters. (D) Multivariable Cox regression for OS using aggregated LAIP categories and known prognostic predictors. LAIPs were included as continuous variables (% of WBCs). (E) Kaplan Meier curve of chromatin-spliceosome class patients stratified by the loss of HLA-DR LAIP (>5% of WBCs).

Beyond our immunophenotypic classification, we investigated whether the abundance of specific LAIPs could provide additional prognostic value beyond genetics. The proportion of cells expressing the loss of HLA-DR LAIP was proportionally associated with inferior OS, even after adjusting for ELN risk (Figure 3D). This LAIP was enriched in two distinct immunophenotypic clusters: the CD34^−^/CD117^+^ promyelocytic-like context (almost exclusively *NPM1*, Pheno1) and within the primitive CD34^+^ compartment (Pheno6) (Figure S6). To further investigate the prognostic value of this immunophenotypic feature, we stratified patients into patients with a pronounced HLA-DR loss LAIP (29.3% of patients) and those without (70.9% of patients) using a binary cut-off (5% of WBCs). This cut-off excludes false positivity by background levels (Figure S3), may include low-level positivity (e.g., low blast counts or subclones), and was set independently of outcomes to avoid overfitting. Interestingly, HLA-DR loss was associated with shorter OS within the chromatin-spliceosome group (Figure 3E), but not within the inv(16), *NPM1*, and *TP53*-aneuploidy groups (all ≥15 HLA-DR loss patients; Figure S10). There were no defining genetic differences between chromatin-spliceosome patients in the HLA-DR-loss high and low groups (Table S7). Together, these findings indicate that HLA-DR loss, potentially associated with immune evasion, is associated with inferior outcomes specifically in patients with chromatin-spliceosome mutations.

### Outcome associations of a lymphocyte-enriched bone marrow diverge on genetic risk

To incorporate the broader immune environment, we included several lymphocyte populations within our patient clustering (Figure 1), identifying a lymphocyte-enriched patient cluster (Pheno5, *n* = 57 patients) consisting of primarily ELN2017 intermediate (*n* = 25) and adverse risk patients (*n* = 22). While proportional increases of lymphocytes could also be attributed to lower blast counts (Figure 1D), we also identified a distinct immunophenotypic cluster with reduced BM blasts but without such lymphocyte enrichment (Pheno4; Figures 1D and 1E). Additionally, lymphocytes were also elevated compared to NBM (Figure 1E). Manual gating of patients in Pheno5 validated low blast counts and enrichment of T, NK, and B cell subsets identified by our computational approach (Figures 4A and 4B), indicating an active inflammatory response within the BM at diagnosis.

**Figure 4:**
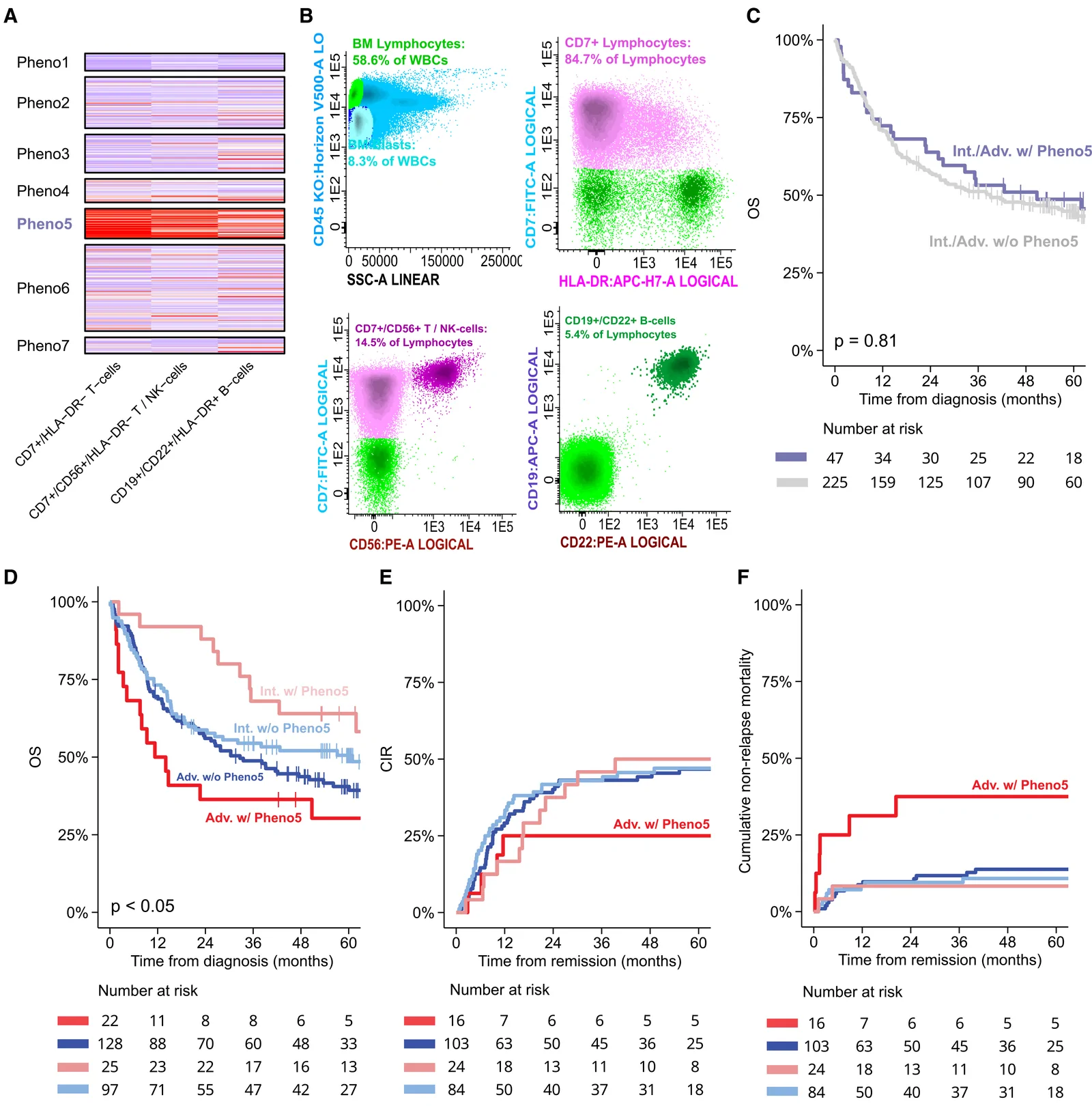
Lymphocyte-enriched bone marrow at diagnosis is related to outcomes in intermediate and adverse risk AML patients (A) Heatmap of lymphoid populations enriched in Pheno5 compared to other patient clusters. Color values are normalized as Z scores. (B) Manual gating of a representative adverse risk patient in Pheno5. (C) Kaplan Meier curve for OS for intermediate/adverse risk patients with or without Pheno5. (D) Kaplan Meier curve for OS split by ELN2017 risk and patient cluster. (E) CIR curve split by ELN risk and patient cluster. (F) Cumulative incidence of non-relapse mortality split by ELN risk and patient cluster. Data are represented as mean ± SD.

We did not observe differences in OS for intermediate/adverse risk patients with and without this characteristic lymphocyte-enriched BM (Pheno5; Figure 4C). Interestingly, stratifying patients by both patient cluster and genetic risk revealed a more complex interaction between lymphocyte enrichment and outcome that was dependent on ELN2017 risk (Figure 4D). Multivariable analysis adjusted for known clinical predictors showed a significant statistical interaction between Pheno5 and ELN2017 risk for OS (Table S8) but not event-free survival (EFS) (Table S9). While we did not observe major mutational differences defining the groups, *FLT3-ITD* was almost absent in intermediate/adverse risk patients with Pheno5 (2/57, 3.5%) compared to other patient clusters (65/225, 28.9%) (Tables S10 and S11). While we did not observe statistically significant differences in the time point of first remission (Tables S12 and S13), 100% of intermediate risk patients with Pheno5 (*n* = 25) reached remission, often after one cycle (76%), compared to 90% and 59% respectively in other patient clusters. Interestingly, adverse risk patients with the lowest OS (Figure 4D) had the lowest Cumulative incidence of relapse (CIR) (Figure 4E) but the highest non-relapse mortality (NRM) (Figure 4F). Taken together, these results suggest that ∼11% of AML patients (mostly intermediate/adverse risk, primarily *FLT3*^wt^) have a BM environment enriched for lymphocyte populations at diagnosis. For patients with adverse risk, this correlates with low relapse rates but increased NRM. For patients with intermediate risk, the lymphocyte-enriched BM environment was associated with high rates of remission after one cycle.

## Discussion

In this study, we applied state-of-the-art computational modeling to map the immunophenotypic characteristics of 502 AML patients at diagnosis, profiling more than 50 million cells across healthy and malignant compartments. Using an unbiased approach, we identified seven distinct immunophenotypic patient clusters. Several of these clusters corresponded closely to known genetic aberrations, including *NPM1* mutations and t(8;21), providing direct evidence for genotype-immunophenotype associations. Additional genetic lesions could be accurately predicted from individual immunophenotypic features, such as specific LAIP or HSPC subsets. For loss of HLA-DR expression on leukemic blasts and the enrichment of lymphocytes within the BM, immunophenotypic features improved patient prognostication in defined genetic contexts. Together, these findings illustrate the extent to which the dynamic and heterogeneous cell-surface landscape of AML and broader BM environment reflect the underlying (epi)genetic characteristics of leukemic cells.

Associations of LAIPs with leukemic blasts have prompted the development of cellular and antibody-based therapies directed against cross-lineage LAIPs such as CD7^11^ and CD19^12^ in AML. However, other approaches have also included myeloid markers such as CD33.^10^ We found robust genotype-phenotype associations in our treatment-naïve population that might be of interest. B, T, and NK cell markers that are frequently overexpressed across different AML cases were enriched explicitly for *CEPBA*^biallelic^ (CD7), inv(16) (CD56), and t(8;21) (CD19, CD56) AMLs, respectively. These subgroups therefore provide important leads for identifying molecular mechanisms driving aberrant cell-surface characteristics. Beyond aberrant cross-lineage expression, many AMLs also show an aberrant lack of expression of markers like CD13, CD33, and HLA-DR. Such patterns allow for therapy-escape of, for instance, CD33-directed therapies such as gemtuzumab ozogamicin or CAR-T therapies. Our comprehensive characterization identified genomic subgroups where such therapies are less likely to succeed, such as *IDH2*^R172^, inv(16), and chromatin-spliceosome subgroups that frequently lacked CD33 expression. For *IDH2*-mutated AML, we find additional evidence supporting further stratification based on the specific locus (R172, R140), as these patients showed distinct immunophenotypic signatures.

Although immunophenotypic patient classification overall was not associated with outcomes following conventional induction and consolidation therapy, we identified several context-specific prognostic markers. In particular, loss of HLA-DR expression on leukemic blasts was associated with inferior outcomes in patients harboring chromatin-spliceosome mutations. Previous studies reported no prognostic impact of HLA-DR loss^29^^,^^30^ and suggested preserved antigen presentation.^30^ However, these cohorts were enriched for *NPM1*-mutated cases with CD34-negative immunophenotype. More recent data have linked preserved HLA-DR expression before transplantation to favorable outcomes.^31^ Our findings, therefore, motivate further investigation into the molecular drivers of HLA-DR downregulation and whether this contributes to immune escape mechanisms. This is of particular interest for patients with chromatin-spliceosome mutations, now all classified as adverse risk in the ELN2022 classification.

We also identified a distinct subgroup comprising ∼11% of patients characterized by lymphocyte enrichment, exceeding levels observed in healthy controls. These cases were almost exclusively *FLT3*^WT^ and demonstrated different clinical trajectories depending on genetic risk. Patients with intermediate-risk disease showed rapid and high rates of remission, whereas those with adverse-risk genetics experienced inferior outcomes, driven in part by increased NRM. These observations suggest that immunophenotyping may provide complementary information to genetic risk stratification by capturing features of antigenicity and the inflammatory state within the BM environment. Longitudinal assessment of such states, from diagnosis through MRD evaluation and the peri-transplant period, warrants further study.

This study is the largest study to date to interrogate the genotype-immunophenotype associations in AML across both malignant and non-malignant cell populations. Although external validation was not feasible, our findings are supported by several related data-driven studies conducted in smaller cohorts (*n* ≤ 122)^7^^,^^23^ or focused on broad myeloid maturation patterns.^6^ Consistent with Couckuyt et al.,^7^ we show with similar high accuracy that immunophenotypic features alone can accurately predict *NPM1* and inv(16) status, supporting the idea that diagnostic flow cytometry can partially anticipate genetic classification. In line with Vergez et al.,^6^ we also identified prognostic associations between specific immunophenotypic features and outcomes among patients receiving intensive treatment. Importantly, the larger scale of our cohort (*n* = 502 AML patients) enabled us to identify additional genotype-immunophenotype association, including at the level of individual loci (e.g., R172 and R140 in *IDH2*-mutated AML), and to place differences in survival within their specific genetic contexts.

In summary, our study demonstrates that large-scale, computational modeling of diagnostic AML samples captures clinically and biologically meaningful heterogeneity that extends beyond established genetic classifications. By integrating both leukemic and non-malignant populations, we reveal immunophenotypic states that may reflect underlying genomic lesions, immune states, and disease biology. As cytometry technologies, and computational methods continue to advance, systematic incorporation of high-dimensional immunophenotyping into clinical and translational studies has the potential to improve risk stratification, identify resistance mechanisms, and inform therapeutic strategies across evolving treatment landscapes in AML and beyond.

### Limitations of the study

Several limitations should be considered. First, while we have previously demonstrated the utility of computational approaches for automated MRD assessment,^32^^,^^33^ we focused here only on diagnostic samples, as the rarity of residual leukemic cells after induction therapy significantly challenges clustering-based analysis. Although we recovered many known LAIPs, some were not detected, potentially due subtle patterns beyond the model’s sensitivity. Conversely, we identified multiple clusters with low proportions in NBM that did not align with expert-defined LAIPs. These “different-from-normal” populations may nonetheless contain leukemic cells but require difficult orthogonal validation to reproduce these high-dimensional aberrancies in another single-cell resolution platform (e.g., FACS). Second, blast counts and broader BM immune compositions confounded relative cell population frequencies, thereby influencing the patient clustering. Restricting our analyses to the canonical CD45dim blast compartment could have mitigated this effect but this would have excluded relevant populations found in monocytic AMLs and is difficult to implement without standardized scatter parameters.^34^ Lastly, while the HOVON-SAKK-132 cohort was sufficiently large to capture robust immunophenotypic characteristics, our analysis remained underpowered for smaller genetic subgroups and did not allow for extensive internal validation. Therefore, external validation in other cohorts, in particular those with different clinical characteristics and treatment regimens, is required.

## Resource availability

### Lead contact

Requests for further information and resources should be directed to the lead contact, Costa Bachas (c.bachas@amsterdamumc.nl).

### Materials availability

• This study did not generate new unique reagents.

### Data and code availability

• Data reported in this paper are available from the lead contact, Costa Bachas, upon reasonable request, although restrictions may apply due to patient privacy and stakeholder approval.
• The MSGMM R package is available open-access for broader use beyond this work on Github: https://github.com/AUMC-HEMA/MSGMM.
• Any additional information required for reproducing the data analysis are available from the lead contact.

## Acknowledgments

The authors thank all participating patients and centers of the Dutch-Belgian Cooperative Trial Group for Hematology-Oncology (HOVON) and Swiss Group for Clinical Cancer Research (SAKK) 132 trial for their contribution.

## Author contributions

T.R.M.: conceptualization, data curation, formal analysis, investigation, methodology, project administration, software, visualization, writing – original draft, writing – review and editing. T.R.: data curation, formal analysis, investigation, methodology, software, visualization, writing – review and editing. L.H.H.: data curation, formal analysis, investigation, writing – review and editing. E.P.: formal analysis, investigation, software. A.K.: formal analysis, investigation, validation. W.J.S.: formal analysis, investigation, validation. D.A.B.: investigation, writing – review and editing. T.F.: investigation, investigation, writing – review and editing. B.T.G.: investigation. L.G.: investigation. G.J.: investigation. J.A.M.: investigation. M.G.M.: investigation. T.P.: investigation. J.R.P.: investigation. K.P.: investigation. J.J.J.: investigation. P.G.: data curation, writing – review and editing. B.L.: investigation. G.J.O.: investigation. A.A.v.d.L.: investigation, writing – review and editing. D.C.d.L.: investigation, writing – review and editing. J.C.: funding acquisition, resources, supervision, writing – review and editing. P.J.V.: investigation, writing – review and editing. C.B.: conceptualization, project administration, supervision, writing – review and editing.

## Declaration of interests

D.A.B.: Sanofi: Consultancy; Daiichi-Sankyo: Consultancy, Honoraria; Takeda: Consultancy, Honoraria; Otsuka: Consultancy: Sobi: Honoraria; Johnson and Johnson: Honoraria; Abbvie: Honoraria; MSD: Honoraria. B.T.G.: BerGenBio: Consultancy; GreinDX: Consultancy; Immedica: Consultancy; InCyte: Consultancy; Mendus AB: Consultancy, Research Funding; Novartis: Consultancy, Research Funding; Otsuka: Consultancy; Pfizer: Consultancy, Research Funding; Sanofi: Consultancy; in Alden Cancer Therapy AS: Current holder of stock options in a privately-held company; KinN Therapeutics AS: Current holder of stock options in a privately-held company; Coegin: Consultancy. L.G.: Miltenyi Biomedicine: Membership on an entity’s Board of Directors or advisory committees. G.J.: AbbVie: Honoraria; Jazz: Honoraria; Laboratoire Delbert: Other: Research cooperation; Novartis: Honoraria; Servier: Honoraria. B.L.: Servier Advisory Board; Syndax Pharmaceuticals Advisory Board; Ryvu Pharmaceuticals Consultant. D.C.d.L.: Takeda: Membership on an entity’s Board of Directors or advisory committees; Abbvie: Consultancy; Roche: Consultancy; Servier: Consultancy, Membership on an entity’s Board of Directors or advisory committees; Ellipses Pharma: Research Funding. A.A.v.d.L.: BMS: Membership on an entity’s Board of Directors or advisory committees, Research Funding; Celgene: Membership on an entity’s Board of Directors or advisory committees, Research Funding; Roche: Research Funding. G.J.O.: Servier: Consultancy; Abbvie: Consultancy; Roche: Consultancy, Membership on an entity’s Board of Directors or advisory committees; Astellas: Consultancy, Honoraria; Gilead: Consultancy; Amgen: Consultancy; AGIOS: Consultancy, Honoraria; Janssen: Novartis and Bristol Myers Squibb: research funding; Incyte: speaker’s fee; AbbVie, Novartis, Pfizer, and Incyte: honoraria (all to institute); president of nonprofit Apps for Care and Science foundation which received unrestricted educational grants from AbbVie, Alexion, Amgen, Astellas, Astra Zeneca, Bristol Myers Squibb, Daiichi Sankyo, Janssen-Cilag, Novartis, Novo-Nordisk, Incyte, Sanofi Genzyme, Servier, Sobi, Jazz, and Takeda; Cloos: Navigate: Consultancy, Patents & Royalties: Royalties MRD assay; BD Biosciences: Patents & Royalties: Royalties LSC tube; Takeda: Research Funding; Novartis: Consultancy, Research Funding.

## STAR★Methods

### Key resources table

**Table:** 

REAGENT or RESOURCE	SOURCE	IDENTIFIER
**Antibodies**		

Anti human CD7-FITC	Becton Dickinson	Cat# 555360; RRID: AB_395763
Anti human CD15-FITC	Becton Dickinson	Cat#332778; RRID: AB_2868627
Anti human CD36-FITC	Becton Dickinson	Cat# 656151; RRID: AB_2870400
Anti human CD2-FITC	Dako Agilent Technologies	Cat# F076701; RRID: AB_578670
Anti human CD45RA-FITC	Becton Dickinson	Cat# 335039; RRID: AB_2868682
Anti human CD56-PE	Becton Dickinson	Cat# 345810; RRID: AB_2868831
Anti human CD22-PE	Becton Dickinson	Cat# 337899; RRID: AB_2868714
Anti human CD14-PE	Becton Dickinson	Cat# 345785; RRID: AB_2868811
Anti human CD133-PE	Miltenyi Biotec	Cat# 130-113-108; RRID: AB_2725937
Anti human Clec12a-PE	Becton Dickinson	Cat# 562566; RRID: AB_11154581
Anti human Tim-3-PE	Becton Dickinson	Cat# 563422; RRID: AB_2716866
Anti human CD7-PE	Becton Dickinson	Cat# 555361; RRID: AB_395764
Anti human CD11b-PE	Becton Dickinson	Cat# 333142; RRID: AB_2868643
Anti human CD22-PE	Becton Dickinson	Cat# 337899; RRID: AB_2868714
Anti human CD34-PerCP-CY5.5	Becton Dickinson	Cat# 347222; RRID: AB_2868843
Anti human CD123-PerCP-CY5.5	Becton Dickinson	Cat# 558714; RRID: AB_1645547
Anti human CD117-Pe-Cy7	Beckman Coulter	Cat# B49221; RRID: AB_2877689
Anti human CD34-Pe-Cy7	Becton Dickinson	Cat# 348811; RRID: AB_2868855
Anti human CD33-APC	Becton Dickinson	Cat# 345800; RRID: AB_2868824
Anti human CD19-APC	Becton Dickinson	Cat# 345791; RRID: AB_2868817
Anti human CD11b-APC	Becton Dickinson	Cat# 333143; RRID: AB_2868644
Anti human CD33-APC	Becton Dickinson	Cat# 345800; RRID: AB_2868824
Anti human CD38-APC	Becton Dickinson	Cat# 345807; RRID: AB_2868829
Anti human HLA-DR	Becton Dickinson	Cat# 641411; RRID: AB_2870307
Anti human CD44-APC-H7	Becton Dickinson	Cat# 560532; RRID: AB_1727482
Anti human CD13-BV421	Becton Dickinson	Cat# 562596; RRID: AB_2737672
Anti human CD33	Becton Dickinson	Cat# 562854; RRID: AB_2737405
Anti human CD45-KromeOrange	Beckman Coulter	Cat# B36294; RRID: AB_2833027
Anti human CD45-HV 500c	Becton Dickinson	Cat# 655873; RRID: AB_2870390

### Experimental model and study participant details

The analysis comprises data from newly diagnosed AML patients enrolled in t phase III trial (HOVON-SAKK-132) treated with intensive chemotherapy with or without the addition of lenalidomide.^35^ Patients (*n* = 502) were selected based on complete and reliable MRD and LSC measurements at diagnosis. The median age was 53 years (IQR, 44–60) and the cohort included 274 males and 228 females. For comparison, we included 20 normal bone marrow (NBM) samples obtained during cardiothoracic surgery, processed using identical flow cytometry protocols during the trial period. Involvement of human participants was approved by the institutional review boards of Amsterdam UMC (#METC-2023.0883) and Erasmus MC (#MEC-2013–539) for patients in the HOVON-SAKK-132 trial. The study followed the Declaration of Helsinki with written informed consent from all patients.

### Method details

#### Genotyping

Molecular analysis was performed with the Illumina TruSight Myeloid Sequencing Panel v1.5 (Illumina, San Diego, CA) to evaluate somatic mutations across 54 commonly mutated genes in myeloid malignancies as reported previously.^35^ Co-mutational patterns were used to define genomic subgroups according to the genomic classification of AML by Papaemmanuil et al. (2016).^27^

#### Flow cytometry

Highly standardized flow cytometry measurements were performed on fresh patient samples collected at diagnosis to establish LAIPs for MRD monitoring (tubes P1-P4) and to identify LSCs (tube P5).^36^ Measurements were performed on a FACS CANTO II (BD Biosciences) using a standardized 5-tube (P1-P5), 8-color panel (Table S1) as described previously.^21^ Patients with less than 10,000 (white blood cell, WBC) events in at least one tube (*n* = 7) were excluded.

#### Flow cytometry pre-processing

Flow cytometry standard (FCS) files were processed using a custom R (v4.3.1) pipeline. First, events exceeding instrument detection capacity were eliminated using PeacoQC (v1.10.0),^37^ followed by fluorescence compensation (flowCore, v2.12.0).^38^ Marker expression levels were transformed using logicle transformations with linearization width (*w*) values determined by the median estimated value across all files per panel using the estimateLogicle function. After transformation, doublets were removed using PeacoQC and flowStats (v4.12.0),^39^ and time-dependent fluorescence anomalies were discarded with PeacoQC. Pre-processed files were inspected for potential anomalies and batch effects using CytoScan (v1.0.0).

#### Unsupervised clustering using multi-sample Gaussian mixture models

Unsupervised clustering was performed for each tube separately based on fluorescence parameters using our previously reported multi-sample Gaussian mixture model (MSGMM).^24^ A maximum of 100,000 cells per file was used for model fitting (Table S2). To maximize detection sensitivity for rare and phenotypically subtle populations, we employed an over-clustering strategy with 100 clusters per panel. After MSGMM clustering, we filtered for CD45-positive WBC clusters. A minimum threshold for the mean of MSGMM cluster was determined, using the myeloid markers CD13 and CD33, and primitive marker CD34 as positive controls to prevent exclusion of CD45-dim blast clusters (Figure S1). Thresholds were determined independently for P5 due to inter-tube fluorochrome differences. Because we captured a variety of WBC populations including lymphocytes, clusters were enumerated relative to all WBCs, generating an N (patients) x N (cluster proportions) matrix for subsequent analysis.

#### Cluster labeling and annotation

Individual WBC clusters were labeled based on the mean fluorescence intensity (MFI) for each marker, categorizing clusters into negative and positive groups (Figure S2), followed by label concatenation (e.g., “MarkerA+/MarkerB-/MarkerC+“). For several markers (CD34, CD117, CD13, CD11b, CD38, CD44), a “dim” label was also used to provide improved context based on expert background knowledge. For some clusters, high expression variance complicated annotation. We therefore excluded the 5% of the most variable clusters for each marker during annotation. Next, we defined sets of marker expression patterns of healthy and LAIP populations (Tables S3 and S4) based on previous characterization^40^ and in-house expertise to match clusters to cell populations. In cases of uncertainty, expert-based visual inspection in Infinicyt 2.0.5b (Cytognos, Salamanca, Spain) was used to validate cluster annotation. For visualization purposes, several matched LAIP patterns (e.g., CD34+/CD13+/CD7+ and CD34+/CD33+/CD7+) were combined into broader categories (e.g., cross-lineage CD7 expression). When LAIPs were identifiable in different tubes, the tube with the lowest background in NBM was used for further analysis.

#### Immunophenotypic patient clustering

Patients were stratified into patient clusters using a consensus clustering approach similar to a previous method used in AML transcriptomic data.^41^ A nearest neighbor graph was created based on WBC cluster proportions and used for community detection using the Leiden algorithm (igraph, v2.1.4).^42^ To obtain robust clusters, clustering was repeated 1,000 times for different seeds and resolution parameters. From these iterations, we created a consensus matrix based on pairwise co-clustering. This consensus matrix was then used for a final patient clustering using hierarchical clustering (Ward’s D2 linkage), using the elbow criterion to identify an optimal number of clusters.

#### Genomic subgroup prediction

Genomic subgroups from Papaemmanuil et al. (2016)^27^ were predicted from immunophenotypic characteristics using binary (one vs. rest) classification based on proportions of aggregated LAIPs and healthy subsets (Tables S3 and S4). We constrained the model to only learn from and select features that were enriched in each target subgroup, rather than depleted features, to ensure direct genotype-phenotype associations. Rare subgroups (inv(3), t(6;9)) and ambiguous subgroups (e.g., “>2 groups”) were excluded from this analysis. For each genomic subgroup, a nonnegative L1-regularized logistic regression model (glmnet v4.1-10)^43^ was used to predict class probabilities for a held-out fold using 10-fold cross-validation. Internal 5-fold cross-validation was used to select the optimal strength of the regularization parameter (λ). Predicted probabilities for the held-out fold were then used to calculate the receiver operating characteristic (ROC) curve. Following performance evaluation, feature selection (non-zero coefficients) was evaluated for the optimal λ using 10-fold cross-validation across the complete cohort. Robustness of the selected features was evaluated across 100 bootstrap iterations by evaluating the selection frequency across randomly selected subsets (80%) of the complete cohort.

### Quantification and statistical analysis

#### Survival analysis

Survival analyses were performed in R (v4.3.1) using the survival (v3.8–3)^44^ and cmprsk (v2.2-12)^45^ packages. Categorical variables were compared using Fisher’s exact test. For survival analysis, overall survival (OS) was defined as time from diagnosis until death, event-free survival (EFS) as time from diagnosis to treatment failure, relapse or death and survival curves were estimated using the Kaplan Meier estimator and compared with the log rank test Univariable and multivariable Cox proportional hazards regression was used to assess hazard ratios and their confidence intervals for OS and EFS. Covariables for multivariable adjustment were selected based on clinical relevance. The proportional hazards assumption of the Cox models was evaluated by Schoenfeld residuals. Cumulative incidence of relapse (CIR) was defined as time of complete remission (CR) or CR with incomplete blood counts (CRi) until relapse with taking death as competing event, cumulative non-relapse mortality (NRM) after allogeneic hematopoietic cell (alloHCT) transplantation was defined as time from CR/CRi until death taking relapse as competing event. Cumulative incidence curves were estimated using the Aalen Johansen estimator and compared using Gray’s test.

## References

[1] The Cancer Genome Atlas Research Network (2013). Genomic and epigenomic landscapes of adult de novo acute myeloid leukemia. N. Engl. J. Med. Overseas. Ed..

[2] Kramer M.H., Zhang Q., Sprung R., Day R.B., Erdmann-Gilmore P., Li Y., Xu Z., Helton N.M., George D.R., Mi Y. (2022). Proteomic and phosphoproteomic landscapes of acute myeloid leukemia. Blood.

[3] Döhner H., Wei A.H., Appelbaum F.R., Craddock C., DiNardo C.D., Dombret H., Ebert B.L., Fenaux P., Godley L.A., Hasserjian R.P. (2022). Diagnosis and management of AML in adults: 2022 recommendations from an international expert panel on behalf of the ELN. Blood.

[4] Lachowiez C.A., Heiblig M., Aspas Requena G., Tavernier-Tardy E., Dai F., Ashango A.B., Peters D.T., Fang J., Kaempf A., Long N., Eide C.A. (2025). Genetic and Phenotypic correlates of clinical outcomes with Venetoclax in Acute Myeloid Leukemia: The GEN-PHEN-VEN study. Blood Cancer Discov..

[5] Zhao L.-P., Dumas-Rivero T., Barette L., Aguinaga L., Cheffai A., Chauvel C., Dal Bello R., Raffoux E., Clappier E., Duchmann M. (2025). Prognostic significance of monocytic-like phenotype in patients with AML treated with venetoclax and azacytidine. Blood Adv..

[6] Vergez F., Largeaud L., Bertoli S., Nicolau M.-L., Rieu J.-B., Vergnolle I., Saland E., Sarry A., Tavitian S., Huguet F. (2022). Phenotypically-defined stages of leukemia arrest predict main driver mutations subgroups, and outcome in acute myeloid leukemia. Blood Cancer J..

[7] Couckuyt A., Van Gassen S., Emmaneel A., Janda V., Buysse M., Moors I., Philippé J., Hofmans J., Kerre T., Saeys Y., Bonte S. (2025). Unraveling genotype–phenotype associations and predictive modeling of outcome in acute myeloid leukemia. Cytometry B Clin. Cytom..

[8] Mazziotta F., Biavati L., Rimando J., Rutella S., Borcherding N., Parbhoo S., Mukhopadhyay R., Chowdhury S., Knaus H.A., Valent P. (2024). CD8+ T-cell differentiation and dysfunction inform treatment response in acute myeloid leukemia. Blood.

[9] Lane A.A. (2020). Targeting CD123 in AML. Clin. Lymphoma Myeloma Leuk..

[10] Liu J., Tong J., Yang H. (2022). Targeting CD33 for acute myeloid leukemia therapy. BMC Cancer.

[11] Gomes-Silva D., Atilla E., Atilla P.A., Mo F., Tashiro H., Srinivasan M., Lulla P., Rouce R.H., Cabral J.M.S., Ramos C.A. (2019). CD7 CAR T cells for the therapy of acute myeloid leukemia. Mol. Ther..

[12] Zhang X., Wang L., Qiao J., Wang S., Wang L., Liu L., Qin J., Chen Z., Huang W., Zheng Y. (2025). Anti-CD19 CAR-T cell therapy in relapsed/refractory t (8; 21) acute myeloid leukemia with aberrant CD19 expression. Front. Immunol..

[13] Kenderian S.S., Ruella M., Shestova O., Klichinsky M., Kim M., Soderquist C., Bagg A., Singh R., Richardson C., Young R. (2017). Targeting CLEC12A with chimeric antigen receptor T cells can overcome the chemotherapy refractoriness of leukemia stem cells. Biol. Blood Marrow Transplant..

[14] van der Schans J.J., Vishwasrao P., Poels R., Antti M., Wang Z., Quik M., van Arkel J., Reuvekamp T., van de Donk N.W.C.J., Themeli M. (2025). Surface downmodulation of TIM3 safeguards healthy cells but not acute myeloid leukemia from CAR T-cell therapy. HemaSphere.

[15] Reuvekamp T., Bachas C., Cloos J. (2024). Immunophenotypic features of early haematopoietic and leukaemia stem cells. Int. J. Lab. Hematol..

[16] Hurwitz C.A., Raimondi S.C., Head D., Krance R., Mirro J.J., Kalwinsky D.K., Ayers G.D., Behm F.G. (1992). Distinctive immunophenotypic features of t (8; 21)(q22; q22) acute myeloblastic leukemia in children. Blood.

[17] Lin L.-I., Chen C.-Y., Lin D.-T., Tsay W., Tang J.-L., Yeh Y.-C., Shen H.L., Su F.H., Yao M., Huang S.Y., Tien H.F. (2005). Characterization of CEBPA mutations in acute myeloid leukemia: most patients with CEBPA mutations have biallelic mutations and show a distinct immunophenotype of the leukemic cells. Clin. Cancer Res..

[18] Davis A.R., Canady B.C., Aggarwal N., Bailey N.G. (2023). Clinicopathologic Features of IDH2 R172–Mutated Myeloid Neoplasms. Am. J. Clin. Pathol..

[19] Heuser M., Freeman S.D., Ossenkoppele G.J., Buccisano F., Hourigan C.S., Ngai L.L., Tettero J.M., Bachas C., Baer C., Béné M.C. (2021). 2021 Update on MRD in acute myeloid leukemia: a consensus document from the European LeukemiaNet MRD Working Party. Blood.

[20] Cloos J., Valk P.J., Thiede C., Döhner K., Roboz G.J., Wood B.L., Walter R.B., Wang S., Wierzbowska A., Wei A.H., Wu D. (2026). 2025 Update on MRD in Acute Myeloid Leukemia: A Consensus Document from the ELN-DAVID MRD Working Party. Blood.

[21] Zeijlemaker W., Kelder A., Cloos J., Schuurhuis G.J. (2019). Immunophenotypic detection of measurable residual (stem cell) disease using LAIP approach in acute myeloid leukemia. Curr. Protoc. Cytom..

[22] Van Gassen S., Callebaut B., Van Helden M.J., Lambrecht B.N., Demeester P., Dhaene T., Saeys Y. (2015). FlowSOM: using self-organizing maps for visualization and interpretation of cytometry data. Cytometry. A..

[23] Levine J.H., Simonds E.F., Bendall S.C., Davis K.L., Amir E-aD., Tadmor M.D., Litvin O., Fienberg H., Jager A., Zunder E. (2015). Data-driven phenotypic dissection of AML reveals progenitor-like cells that correlate with prognosis. Cell.

[24] Rutten P., Mocking T.R., Cloos J., van Wieringen W.N., Bachas C. (2025). Computationally efficient multi-sample flow cytometry data analysis using Gaussian mixture models. BMC Bioinf..

[25] Weber L.M., Robinson M.D. (2016). Comparison of clustering methods for high-dimensional single-cell flow and mass cytometry data. Cytometry A.

[26] Nowicka M., Krieg C., Crowell H.L., Weber L.M., Hartmann F.J., Guglietta S., Becher B., Levesque M.P., Robinson M.D. (2017). CyTOF workflow: differential discovery in high-throughput high-dimensional cytometry datasets. F1000Res..

[27] Papaemmanuil E., Gerstung M., Bullinger L., Gaidzik V.I., Paschka P., Roberts N.D., Potter N.E., Heuser M., Thol F., Bolli N. (2016). Genomic classification and prognosis in acute myeloid leukemia. N. Engl. J. Med..

[28] Mason E.F., Kuo F.C., Hasserjian R.P., Seegmiller A.C., Pozdnyakova O. (2018). A distinct immunophenotype identifies a subset of NPM1-mutated AML with TET2 or IDH1/2 mutations and improved outcome. Am. J. Hematol..

[29] Wetzler M., McElwain B.K., Stewart C.C., Blumenson L., Mortazavi A., Ford L.A., Slack J.L., Barcos M., Ferrone S., Baer M.R. (2003). HLA-DR antigen-negative acute myeloid leukemia. Leukemia.

[30] Roerden M., Märklin M., Salih H.R., Bethge W.A., Klein R., Rammensee H.-G., Nelde A., Walz J.S. (2021). Expression levels of HLA-DR in acute myeloid leukemia: implications for antigenicity and clinical outcome. Leuk. Lymphoma.

[31] Röhnert M.A., Löffler C., Kramer M., Röllig C., Schetelig J., Stölzel F., Kunadt D., Middeke J.M., Thielecke J., Uschner F., Glauche I. (2025). Prognostic Value of Multicolor-Flow Cytometric Measurable Residual Disease Detection in Acute Myeloid Leukemia Prior to Allogeneic Hematopoietic Cell Transplantation: Potential Role of HLA-DR. Clin. Lymphoma Myeloma Leuk..

[32] Mocking T.R., Kelder A., Reuvekamp T., Ngai L.L., Rutten P., Gradowska P., van de Loosdrecht A.A., Cloos J., Bachas C. (2024). Computational assessment of measurable residual disease in acute myeloid leukemia using mixture models. Commun. Med..

[33] Mocking T.R., Haaksma L.H., Reuvekamp T., Kelder A., Scholten W.J., Ngai L.L., Breems D.A., Fischer T., Gjertsen B.T., Griškevičius L., Juliusson G. (2025). Computational measurable residual disease assessment in acute myeloid leukemia: a retrospective validation in the HOVON-SAKK-132 trial: MINIMAL RESIDUAL DISEASE. Leukemia.

[34] Mocking T.R., van de Loosdrecht A.A., Cloos J., Bachas C. (2025). Applications of machine learning for immunophenotypic measurable residual disease assessment in acute myeloid leukemia. HemaSphere.

[35] Löwenberg B., Pabst T., Maertens J., Gradowska P., Biemond B.J., Spertini O., Vellenga E., Griskevicius L., Tick L.W., Jongen-Lavrencic M. (2021). Addition of lenalidomide to intensive treatment in younger and middle-aged adults with newly diagnosed AML: the HOVON-SAKK-132 trial. Blood Adv..

[36] Cloos J., Harris J.R., Janssen J.J.W.M., Kelder A., Huang F., Sijm G., Vonk M., Snel A.N., Scheick J.R., Scholten W.J. (2018). Comprehensive protocol to sample and process bone marrow for measuring measurable residual disease and leukemic stem cells in acute myeloid leukemia. J. Vis. Exp..

[37] Emmaneel A., Quintelier K., Sichien D., Rybakowska P., Marañón C., Alarcón-Riquelme M.E., Van Isterdael G., Van Gassen S., Saeys Y. (2022). PeacoQC: peak-based selection of high quality cytometry data. Cytometry. A..

[38] Hahne F., LeMeur N., Brinkman R.R., Ellis B., Haaland P., Sarkar D., Spidlen J., Strain E., Gentleman R. (2009). flowCore: a Bioconductor package for high throughput flow cytometry. BMC Bioinf..

[39] Hahne F., Gopalakrishnan N., Khodabakhshi A.H., Wong C., Lee K. (2009). flowStats: Statistical methods for the analysis of flow cytometry data. R Packag version.

[40] Terwijn M., van Putten W.L.J., Kelder A., van der Velden V.H.J., Brooimans R.A., Pabst T., Maertens J., Boeckx N., de Greef G.E., Valk P.J.M. (2013). High prognostic impact of flow cytometric minimal residual disease detection in acute myeloid leukemia: data from the HOVON/SAKK AML 42A study. J. Clin. Oncol..

[41] Severens J.F., Karakaslar E.O., van der Reijden B.A., Sánchez-López E., van den Berg R.R., Halkes C.J.M., van Balen P., Veelken H., Reinders M.J.T., Griffioen M., van den Akker E.B. (2024). Mapping AML heterogeneity-multi-cohort transcriptomic analysis identifies novel clusters and divergent ex-vivo drug responses. Leukemia.

[42] Csardi G., Nepusz T. (2006). The igraph software. Complex Syst..

[43] Friedman J., Hastie T., Tibshirani R., Narasimhan B., Tay K., Simon N., Qian J., Yang J., Taylor J. (2021). Package ‘glmnet’. CRAN R Repositary.

[44] Therneau T. (2015). A package for survival analysis in R. R package version.

[45] Fine J.P., Gray R.J. (1999). A proportional hazards model for the subdistribution of a competing risk. J. Am. Stat. Assoc..

