# Supplementary material for: The immunophenotypic landscape of newly diagnosed acute myeloid leukemia: Associations with genotype and outcomes
Source: iScience. 2026 Oct 12;29(11):117762. doi: 10.1016/j.isci.2026.117762 (PMC13682389; doi:10.1016/j.isci.2026.117762)
Supplement: Supplementary file 1 [file mmc1.pdf]

iScience, Volume *29*

Supplemental information

The immunophenotypic landscape of newly diagnosed acute myeloid leukemia: Associations with genotype and outcomes

Tim R. Mocking, Tom Reuvekamp, Lukas H. Haaksma, Eirini Papavasileiou, Angèle Kelder, Willemijn J. Scholten, Dimitri A. Breems, Thomas Fischer, Bjørn T. Gjertsen, Laimonas Griškevičius, Gunnar Juliusson, Johan A. Maertens, Markus G. Manz, Thomas Pabst, Jakob R. Passweg, Kimmo Porkka, Jeroen J.W.M. Janssen, Patrycja Gradowska, Bob Löwenberg, Gert J. Ossenkoppele, Arjan A. van de Loosdrecht, David C. de Leeuw, Jacqueline Cloos, Peter J.M. Valk, and Costa Bachas

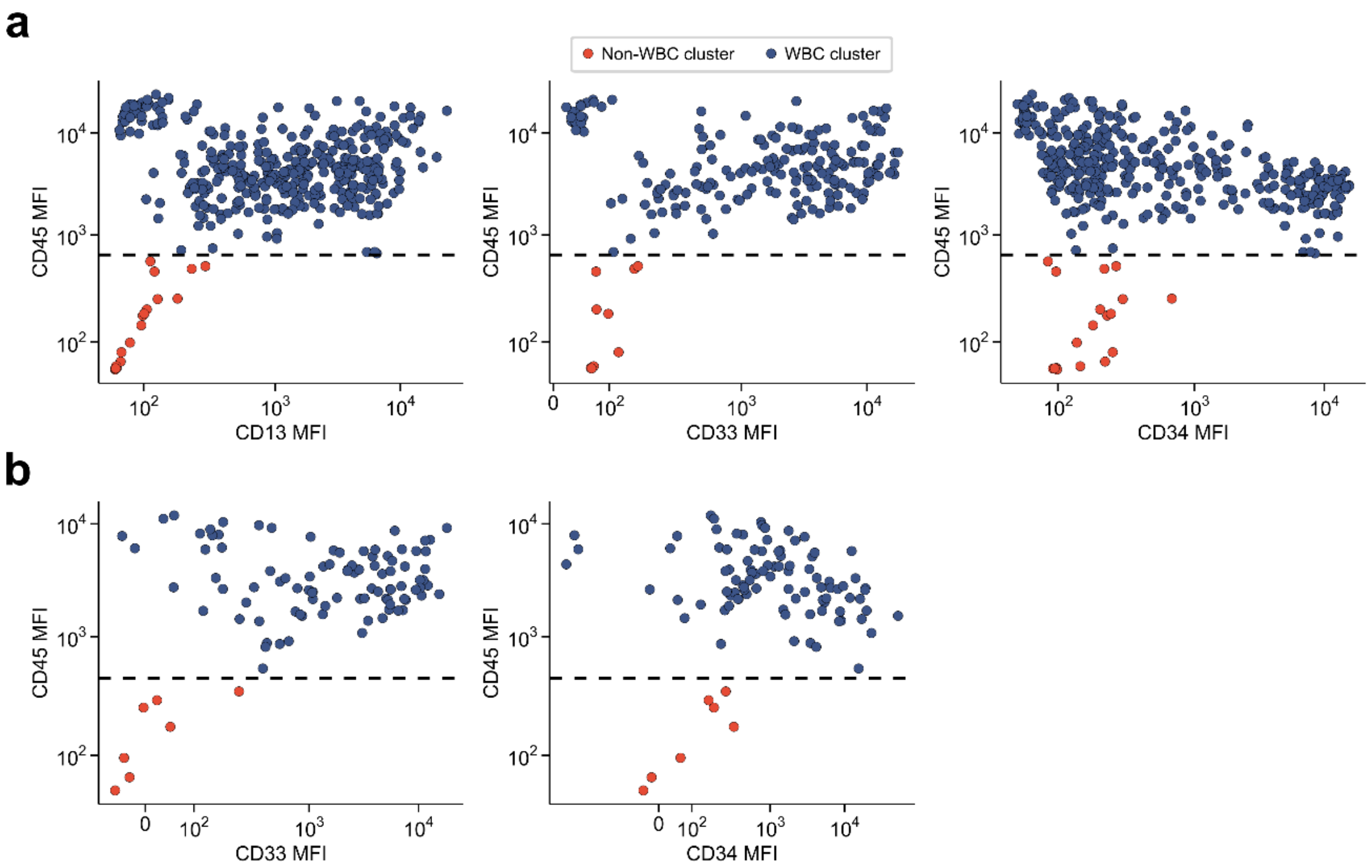

Figure S1. Identification of CD45-expressing WBC clusters based on cluster mean fluorescent intensity (MFI). a CD13, CD33 and CD34 were used as positive controls in MRD tubes (P1-P4). **b** CD34 and CD33 were used in P5. Analyses were performed separately in P1-P4 and P5 due to fluorochrome differences.

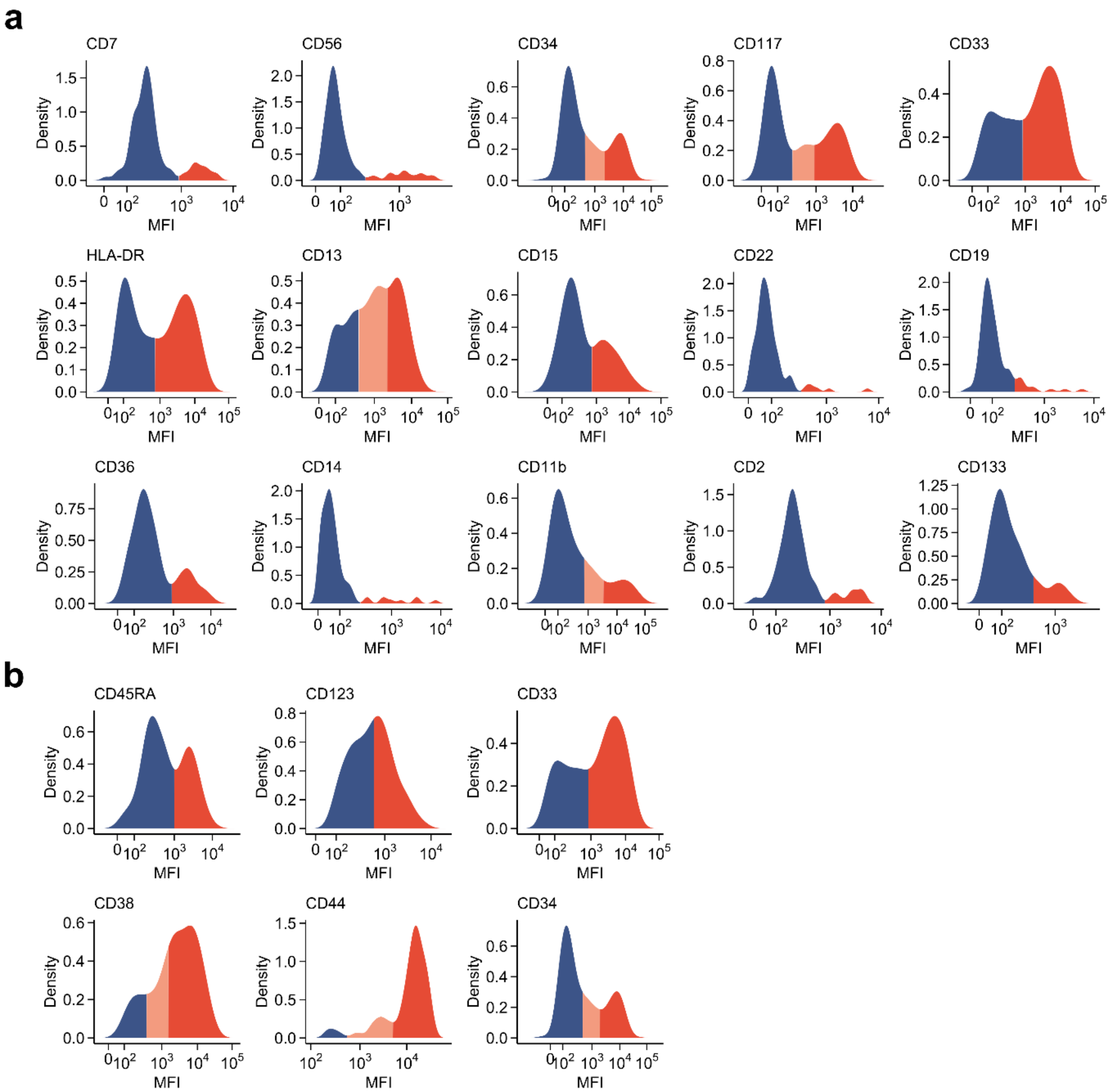

Figure S2. Cluster annotation using marker-based MFI thresholding. a CD13, CD33 and CD34 were used as positive controls in MRD tubes (P1-P4). **b** CD34 and CD33 were used in P5. Analyses were performed separately due to fluorochrome differences.

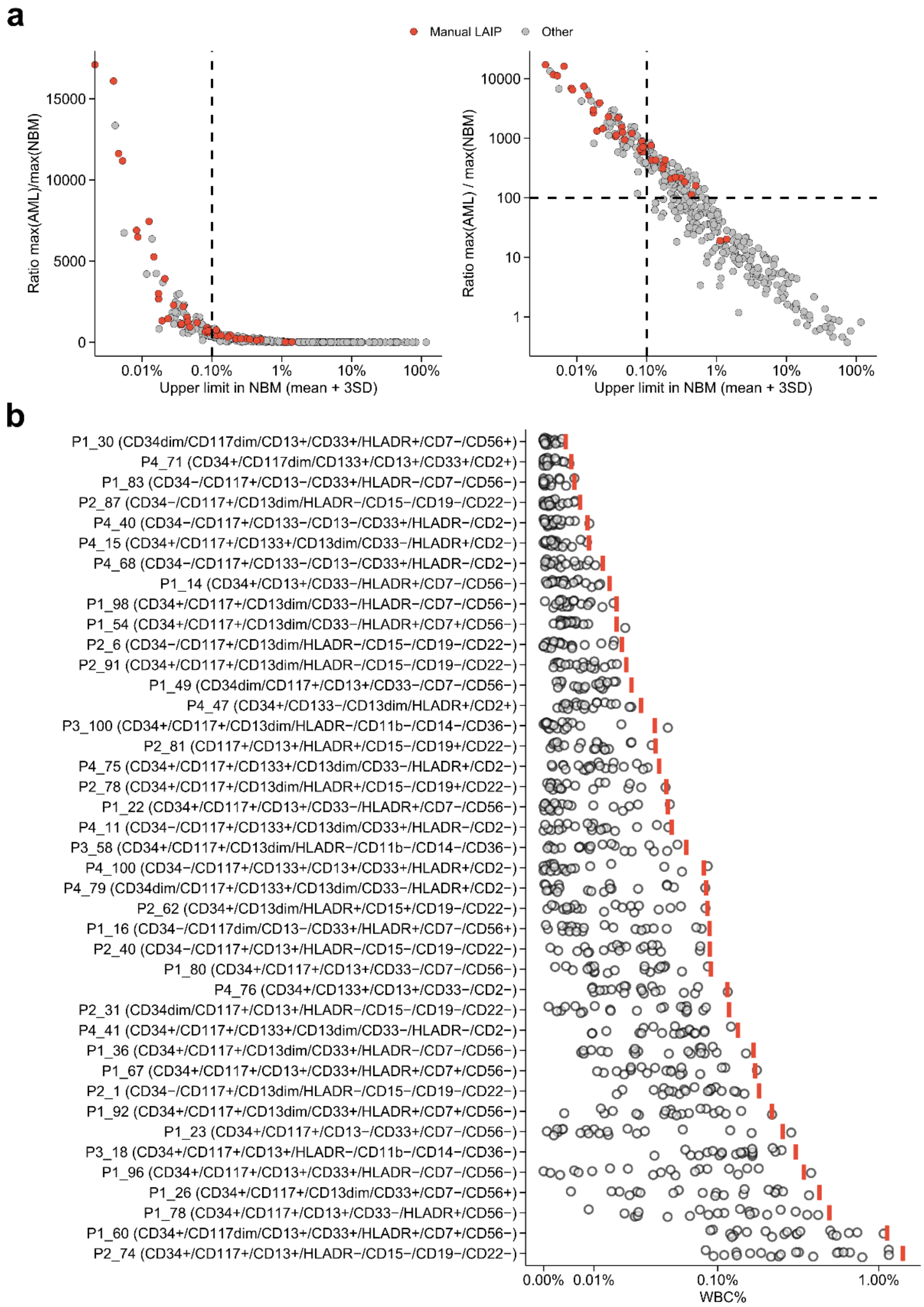

Figure S3. Cluster abundance of potentially leukemic clusters in NBM. a NBM background levels per cluster. **b** NBM background levels for known LAIPs for each NBM sample (n=20) separately. Red lines indicate upper limit in NBM (mean + 3 SD).

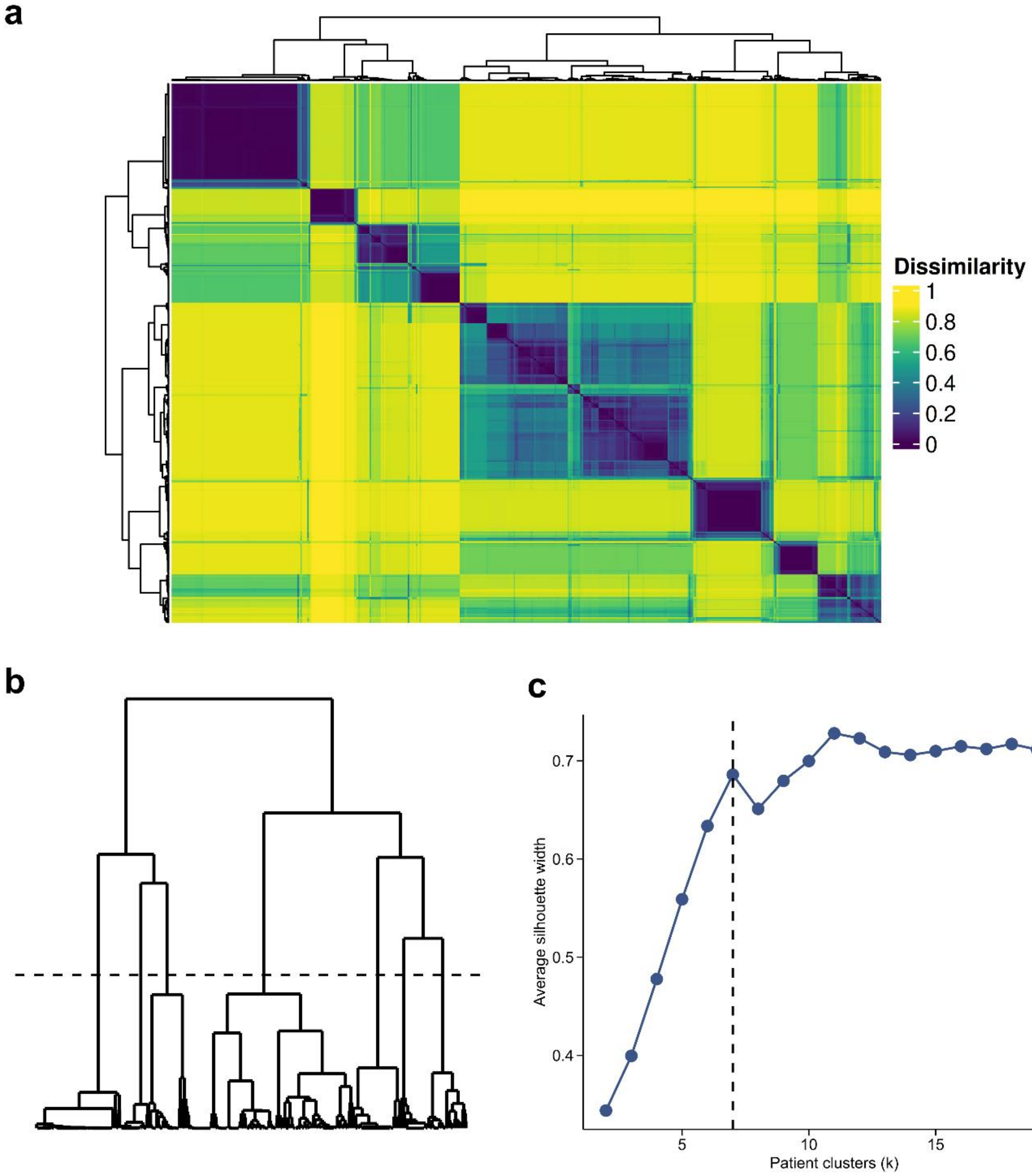

Figure S4. Consensus clustering of patients using the Leiden algorithm. a Dissimilarity matrix (1 – consensus) generated across 1,000 iterations of the Leiden algorithm. **b** Hierarchical clustering of dissimilarity matrix (ward.d2). Dotted line indicates selected patient clustering resolution (k=7). **c** Silhouette scores for hierarchical clustering of dissimilarity matrix.

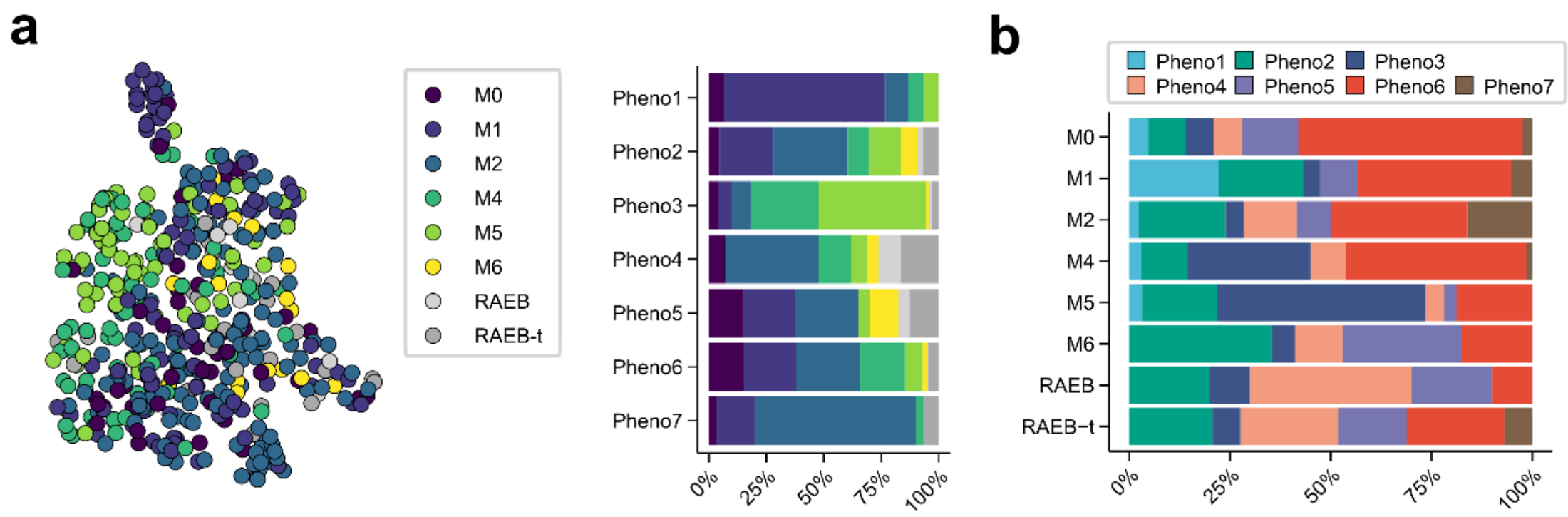

Figure S5. Association between immunophenotypic clustering and FAB classification. a UMAP of cluster proportions colored by FAB classification. Bar plot specifies proportions of FAB classes per immunophenotypic cluster. **b** Proportions of immunophenotypic clusters per FAB group.

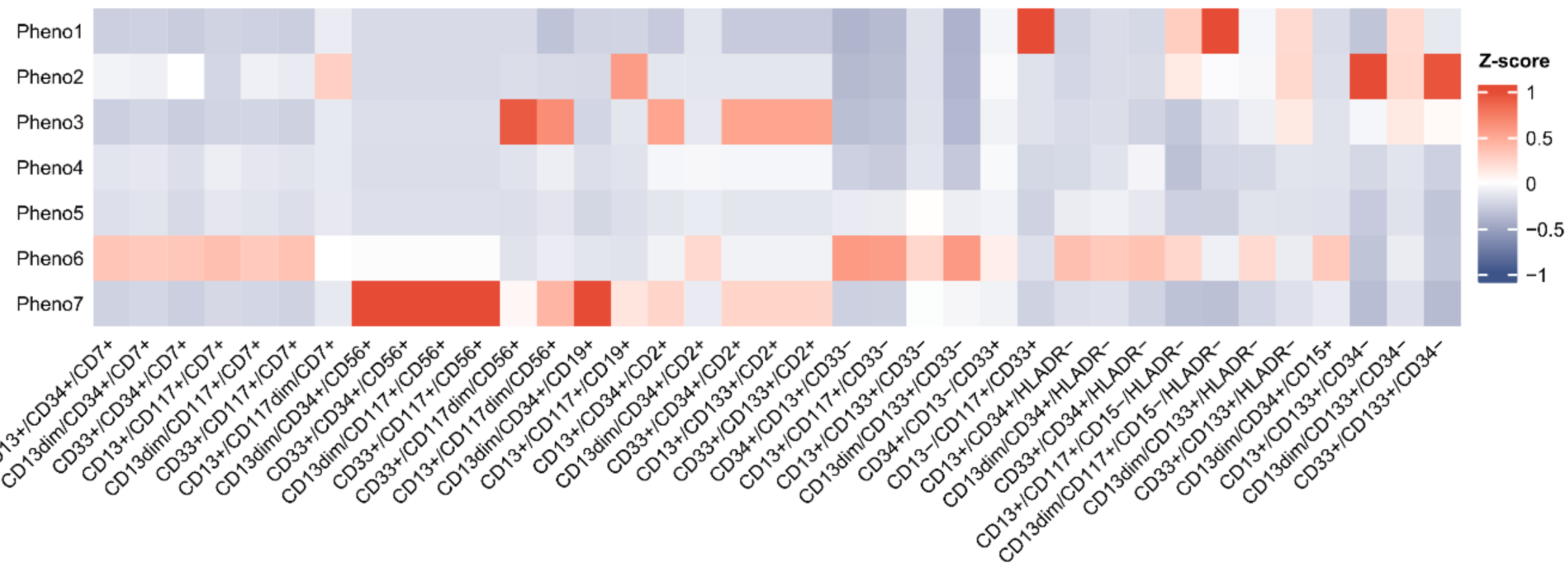

Figure S6. Heatmap of unaggregated LAIPs per patient cluster.

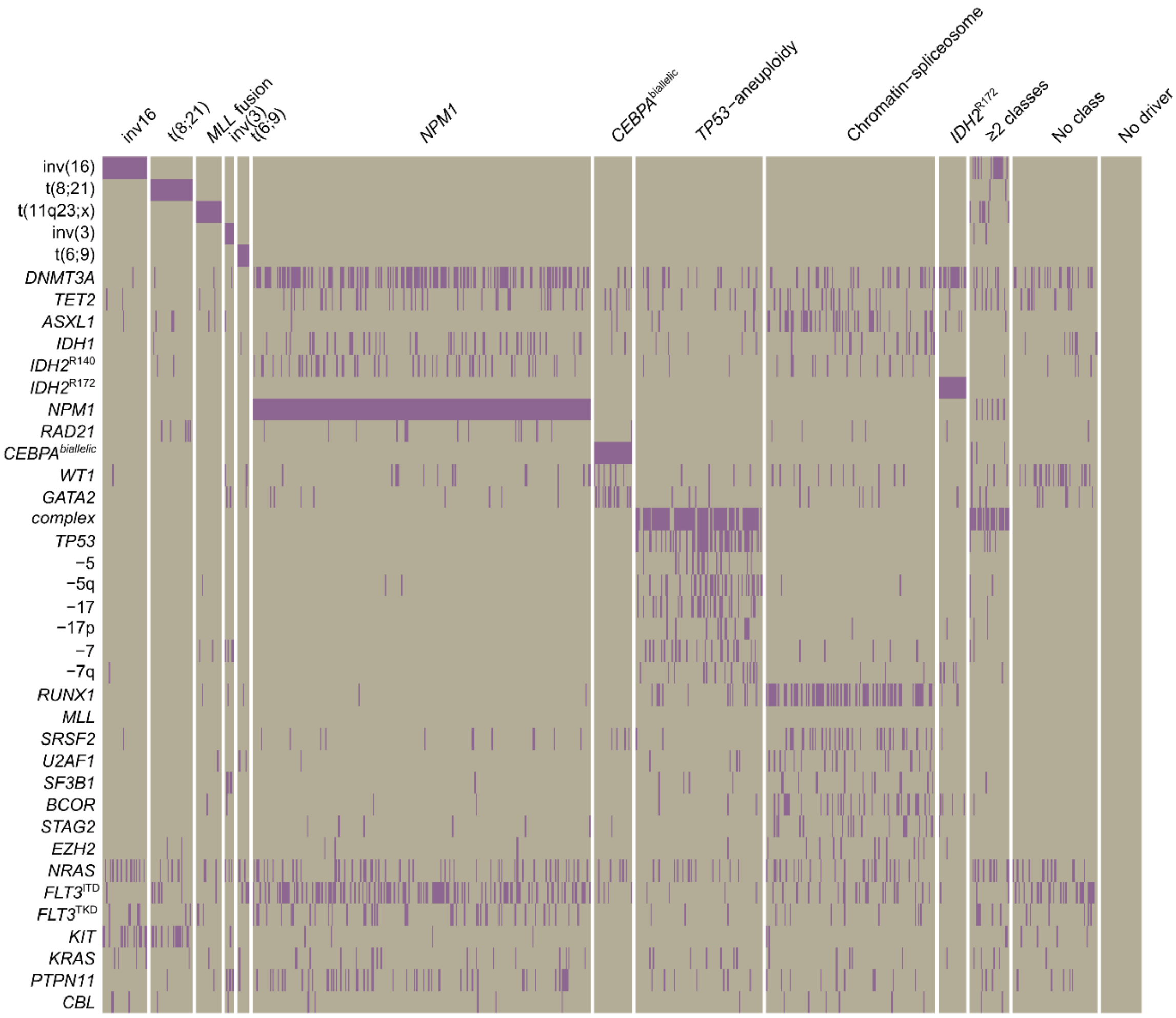

Figure S7. Co-occurring patterns of genetic lesions for AML patients stratified by genomic class in the HOVON-SAKK-132 trial. Only selected mutations are shown for visual clarity based on earlier reporting in Papaemmanuil *et al*.

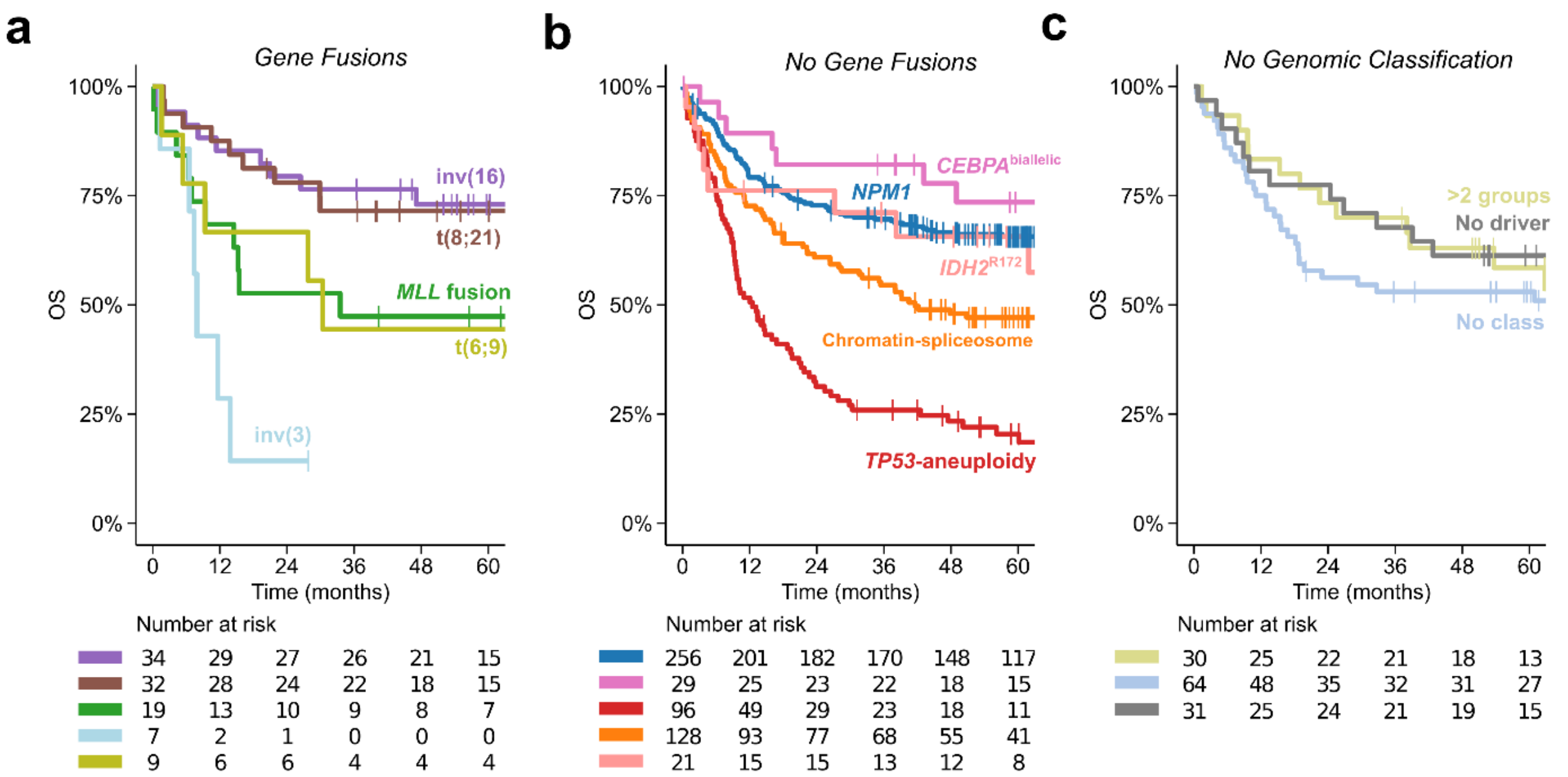

Figure S8. Kaplan Meier curves for overall survival in the HOVON-SAKK-132 trial stratified by genomic classes.

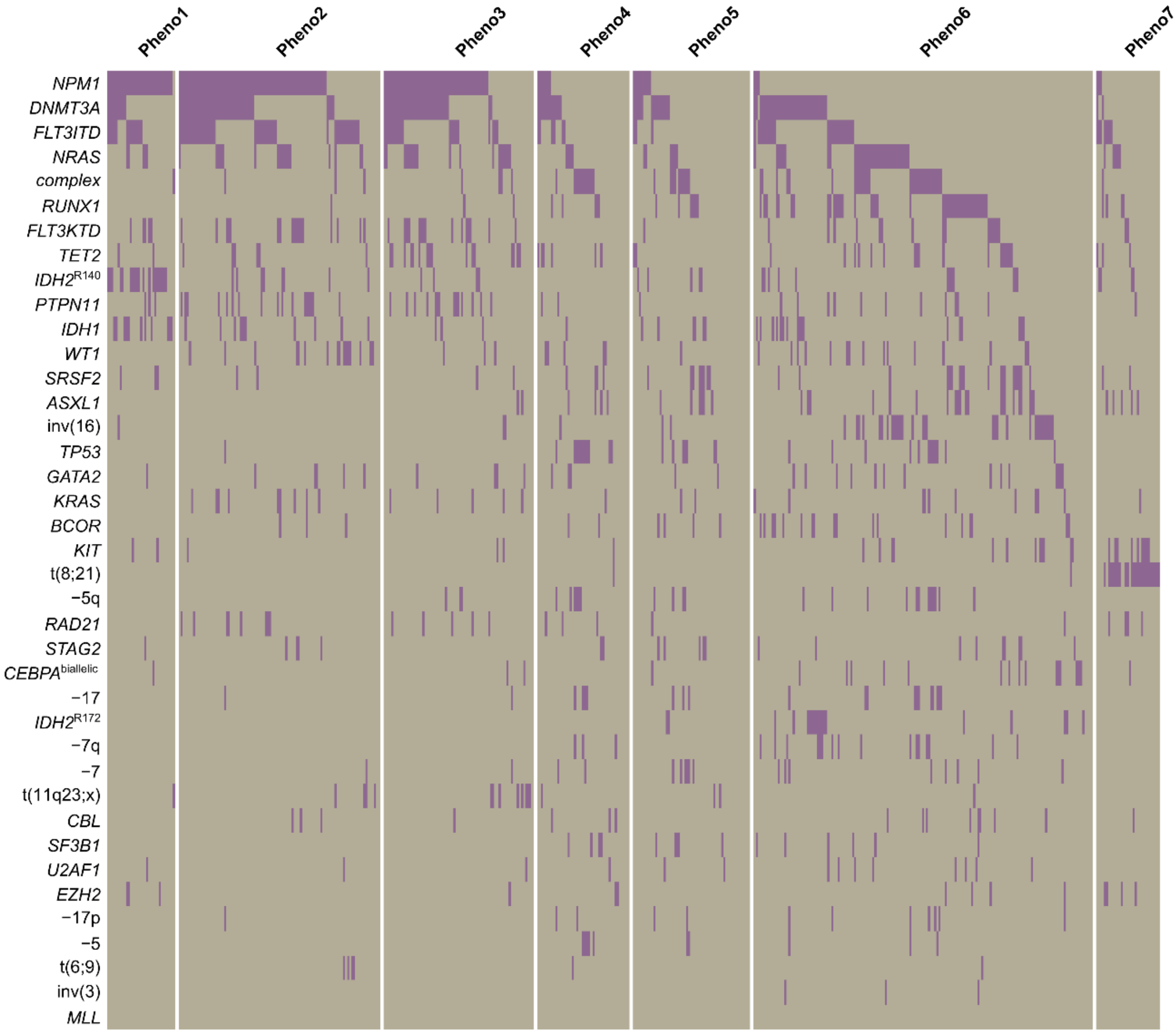

Figure S9. (Co-)mutational landscape of genetic lesions across immunophenotypic patient clusters.

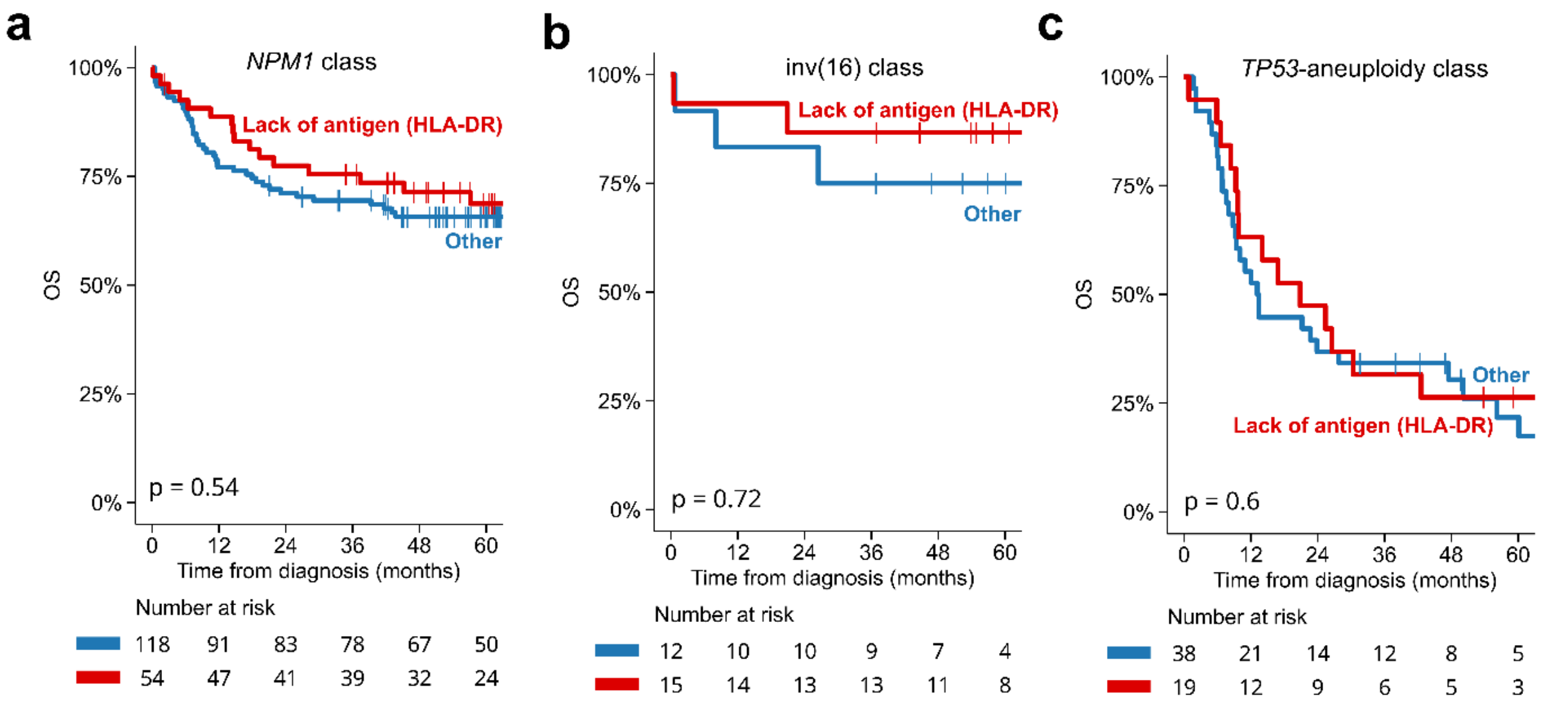

Figure S10. Kaplan Meier curves for overall survival stratified by genomic classes and lack of HLA-DR expression.

Table S1. Flow cytometry panel used for measurable residual disease and leukemia stem cell assessment at diagnosis timepoint in HOVON-SAKK-132 trial.

Channel	P1	P2	P3	P4	P5
**FITC**	CD7	CD15	CD36	CD2	CD45RA
**PE**	CD56	CD22	CD14	CD133	COMBI*
**PerCP-CY5.5**	CD34	CD34	CD34	CD34	CD123
**PC7**	CD117	CD117	CD117	CD117	CD33
**APC**	CD33	CD19	CD11b	CD33	CD38
**APC-H7**	HLA-DR	HLA-DR	HLA-DR	HLA-DR	CD44
**BV421**	CD13	CD13	CD13	CD13	CD34
**KO**	CD45	CD45	CD45	CD45	CD45

*The COMBI channel (CLEC12A, CD56, CD7, CD11b, TIM-3 and CD22) was excluded from clustering.

Table S2. Number of cells evaluated by MSGMM for clustering.

Tube	# Training cells	# Cells evaluated
**P1**	50,979,352	77,060,202
**P2**	50,870,376	78,893,543
**P3**	50,935,628	75,001,575
**P4**	51,044,808	73,639,578
**P5**	53,030,863	1,366,791,782

Note: Training cells refers to a limited number of cells used for clustering (model fitting), while all cells were evaluated after model fitting to calculate cluster proportions.

Table S3. Pattern matching of MSGMM component labeling to known cell populations.

Marker pattern(s)	Assigned population
CD34+/CD38dim/CD45RA-/CD123-/CD44+	HSCs
CD45RA+/CD123+/CD34+/CD38+	GMPs
CD45RA-/CD123+/CD34+/CD38+/CD44+	CMPs
CD45RA-/CD123-/CD38+/CD44+/CD33-	MEPs
CD34-/CD117+/CD13+/HLADR+/CD11b-/CD14-/CD36-<br>CD34-/CD117dim/CD13+/HLADR+/CD11b-/CD14-/CD36-	Monoblasts
CD34-/CD117-/CD13dim/HLADR+/CD11bdim/CD14-/CD36+	Promonocytes
CD34-/CD117-/CD13+/HLADR+/CD11b+/CD14-/CD36+<br>CD34-/CD117-/CD13dim/HLADR+/CD11b+/CD14-/CD36+	CD11b+/CD14-/CD36+ (Pro)monoyctes
CD34-/CD117-/CD13+/HLADR+/CD11b+/CD14+/CD36+<br>CD34-/CD117-/CD13dim/HLADR+/CD11b+/CD14+/CD36+	CD11b+/CD14+/CD36+ Monocytes
CD34-/CD117+/CD13+/HLADR-/CD11b-/CD14-/CD36-<br>CD34-/CD117+/CD13dim/HLADR-/CD11b-/CD14-/CD36-<br>CD34-/CD117+/CD13+/HLADR-/CD11bdim/CD14-/CD36-<br>CD34-/CD117+/CD13dim/HLADR-/CD11bdim/CD14-/CD36-	Promyelocytes
CD34-/CD117-/CD13dim/HLADR-/CD11b+/CD14-/CD36-<br>CD34-/CD117-/CD13dim/HLADR-/CD11bdim/CD14-/CD36-	(Meta)myelocytes
CD34-/CD117-/CD13-/CD33-/HLADR-/CD7+/CD56-<br>CD34-/CD117-/CD13-/CD33-/HLADR+/CD7+/CD56-	CD7+/HLA-DR- T-cells
CD34-/CD117-/CD13-/CD33-/HLADR-/CD7+/CD56+<br>CD34-/CD117-/CD13-/CD33-/HLADR+/CD7+/CD56+	CD7+/CD56+/HLA-DR- T / NK-cells
CD34-/CD117-/CD13-/HLADR+/CD15-/CD19+/CD22+<br>CD34-/CD117-/CD13-/HLADR-/CD15-/CD19+/CD22+	CD19+/CD22+/HLA-DR+ B-cells
CD34-/CD117-/CD13-/HLADR+/CD11b-/CD14-/CD36+	Dendritic cells

Table S4. Pattern matching of Gaussian component labeling to known LAIP populations.

Marker pattern(s)	Aggregated category
CD13+/CD34+/CD7+<br>CD13dim/CD34+/CD7+<br>CD33+/CD34+/CD7+<br>CD13+/CD117+/CD7+<br>CD13dim/CD117+/CD7+<br>CD33+/CD117+/CD7+<br>CD13+/CD117dim/CD7+	Cross-lineage (CD7)
CD13+/CD34+/CD56+<br>CD13dim/CD34+/CD56+<br>CD33+/CD34+/CD56+<br>CD13+/CD117+/CD56+<br>CD13dim/CD117+/CD56+<br>CD33+/CD117+/CD56+<br>CD33+/CD117dim/CD56+<br>CD13+/CD117dim/CD56+	Cross-lineage (CD56)
CD13+/CD34+/CD19+<br>CD13dim/CD34+/CD19+<br>CD13+/CD117+/CD19+	Cross-lineage (CD19)
CD13+/CD34+/CD22+<br>CD13dim/CD34+/CD22+<br>CD13+/CD117+/CD22+	Cross-lineage (CD22)
CD13+/CD34+/CD2+<br>CD13dim/CD34+/CD2+<br>CD33+/CD34+/CD2+<br>CD13+/CD117+/CD2+<br>CD33+/CD117+/CD2+<br>CD13+/CD133+/CD2+<br>CD33+/CD133+/CD2+	Cross-lineage (CD2)
CD34+/CD13+/CD33-<br>CD13+/CD117+/CD33-<br>CD13+/CD133+/CD33-<br>CD13dim/CD133+/CD33-	Lack of antigen (CD33)
CD34+/CD13-/CD33+<br>CD13-/CD117+/CD33+<br>CD13-/CD33+/CD133+	Lack of antigen (CD13)
CD13+/CD34+/HLADR-<br>CD13dim/CD34+/HLADR-<br>CD33+/CD34+/HLADR-<br>CD13+/CD117+/CD15-/HLADR-<br>CD13dim/CD117+/CD15-/HLADR-<br>CD13+/CD117dim/CD15-/HLADR-<br>CD13+/CD133+/HLADR-<br>CD13dim/CD133+/HLADR-<br>CD33+/CD133+/HLADR-	Lack of antigen (HLA-DR)
CD13+/CD34+/CD11b+<br>CD13dim/CD34+/CD11b+<br>CD13+/CD117+/CD11b+<br>CD13dim/CD117+/CD11b+	Asynchronous antigen (CD11b)
CD13dim/CD34+/CD15+	Asynchronous antigen (CD15)
CD13+/CD133+/CD34-<br>CD13dim/CD133+/CD34-<br>CD33+/CD133+/CD34-	Lack of antigen (CD34)
CD13+/CD34+/CD14+/CD36-<br>CD13dim/CD34+/CD14+/CD36-	Asynchronous antigen (CD14)
CD33+/CD34-/CD117-/HLADR+/CD7+<br>CD13+/CD34-/CD117-/HLADR+/CD7+	Cross-lineage (CD7) – Mature
CD13+/CD34-/CD117-/CD14+/CD36+/CD11b-<br>CD13dim/CD34-/CD117-/CD14+/CD36+/CD11b-<br>CD34-/CD117-/CD14+/CD11b-	Asynchronous antigen (CD11b/CD14) - Mature

Table S5. Genomic class assignment in the HOVON-SAKK-132 trial closely resembles the distribution from Papaemmanuil *et al*. in NEJM.

Group	NEJM<br>*no. of patients (%)*	HOVON-SAKK-132<br>*no. of patients (%)*
AML with *NPM1* mutation	418 (28)	256 (34)
AML with mutated chromatin, RNA-splicing genes or both	275 (19)	128 (17)
AML with *TP53* mutations, chromosomal aneuploidy, or both	199 (13)	96 (13)
AML with inv(16)(p13.1q22) or t(16;16)(p13.1q22); *CBFB-MYH11*	81 (5)	34 (4)
AML with biallelic *CEBPA* mutations	66 (4)	29 (4)
AML with t(8;21)(q22;q22); *RUNX1-RUNX1T1*	60 (4)	32 (4)
AML with *MLL fusion* genes; t(x;11) (x;q23)	44 (3)	19 (3)
AML with inv(3)(q21q26.2) or t(3;3)(q21;q26.2); *GATA2*, *MECOM(EVI1)*	20 (1)	7 (1)
AML with $IDH2^{R172}$ mutations and no other class-defining lesions	18 (1)	21 (3)
AML with t(6;9) (p23;q34); *DEK-NUP214*	15 (1)	9 (1)
AML with driver mutations but no detected class-defining lesions	166 (11)	64 (8)
AML with no detected driver mutations	62 (4)	31 (4)
AML meeting criteria for ≥2 genomic subgroups	56 (4)	30 (4)

Proportions from the HOVON-SAKK-132 study are reported based on AML patients in the full clinical cohort with complete molecular diagnostics. t(15;17)(q22;q12); *PML-RARA* was excluded from NEJM for comparison as these patients were not enrolled in HOVON-SAKK-132.

Table S6. Feature selection stability for genomic subgroup prediction.

Genomic subgroup	Immunophenotypic feature	Selected (%)
CEBPA ^{biallelic}	CD13+/CD117+/CD7+	98%
	CD33+/CD117+/CD7+	91%
	GMPs	96%
**Chromatin-spliceosome**	CD34+/CD13+/CD33-	100%
	CD7+/HLA-DR- T-cells	87%
	CMPs	74%
	Dendritic cells	43%
	GMPs	69%
IDH2 $^{\text{R172}}$	CD13+/CD117+/CD33-	83%
	CD13dim/CD133+/CD33-	98%
***MLL* fusion**	CD11b+/CD14-/CD36+ (Pro)monoyctes	74%
	CD33+/CD117dim/CD56+	100%
NPM1	CD11b+/CD14+/CD36+ Monocytes	100%
	CD11b+/CD14-/CD36+ (Pro)monoyctes	100%
	CD13+/CD117dim/CD7+	92%
	CD13+/CD133+/CD34-	60%
	CD13-/CD117+/CD33+	100%
	Monoblasts	100%
	Promonocytes	98%
	Promyelocytes	100%
***TP53*-aneuploidy**	(Meta)myelocytes	79%
	CD13dim/CD34+/CD15+	81%
	CD13dim/CD34+/CD56+	42%
	CD19+/CD22+/HLA-DR+ B-cells	72%
	CD7+/CD56+/HLA-DR- T / NK-cells	47%
	CD7+/HLA-DR- T-cells	66%
	CMPs	100%
**inv(16)**	CD13+/CD117dim/CD56+	85%
	CD33+/CD34+/HLADR-	91%
	CD34+/CD13+/CD33-	37%
	GMPs	96%
**t(8;21)**	CD13dim/CD34+/CD19+	100%
	CD13dim/CD34+/CD56+	100%

Table S7. Genetic differences between Chromatin-spliceosome patients with and without lack of HLA-DR expression.

**Characteristic**	**Overall** N = 86	**Other** N = 71	**Loss of HLA-DR** N = 15	**p-value**
RUNX1	47 (55%)	38 (54%)	9 (60%)	0.8
ASXL1	27 (31%)	24 (34%)	3 (20%)	0.4
SRSF2	26 (30%)	23 (32%)	3 (20%)	0.5
BCOR	18 (21%)	12 (17%)	6 (40%)	0.075
FLT3-ITD	15 (17%)	12 (17%)	3 (20%)	0.7
DNMT3A	13 (15%)	9 (13%)	4 (27%)	0.2
IDH1	13 (15%)	8 (11%)	5 (33%)	0.046
NRAS	13 (15%)	12 (17%)	1 (6.7%)	0.4
STAG2	13 (15%)	10 (14%)	3 (20%)	0.7
IDH2	12 (14%)	9 (13%)	3 (20%)	0.4
SF3B1	9 (10%)	9 (13%)	0 (0%)	0.3
TET2	9 (10%)	9 (13%)	0 (0%)	0.3
U2AF1	9 (10%)	7 (9.9%)	2 (13%)	0.7
EZH2	7 (8.1%)	7 (9.9%)	0 (0%)	0.3
FLT3-TKD	6 (7.0%)	6 (8.5%)	0 (0%)	0.6
WT1	6 (7.0%)	5 (7.0%)	1 (6.7%)	>0.9
GATA2	5 (5.8%)	5 (7.0%)	0 (0%)	0.6

P-values calculated with Fisher's exact test. Only lesions with more than 5 occurrences are shown.

Table S8. Multivariable Cox regression for overall survival.

Characteristic	N	HR	95% CI	p-value
**Age**				
Age	501	1.02	1.01, 1.04	0.001
**Disease type**				
De Novo (reference)	468	—	—	
Secondary	15	1.50	0.76, 2.98	0.25
Treatment-related	18	1.31	0.69, 2.50	0.41
**WBC**				
WBC	501	1.00	1.00, 1.00	0.76
**Patient cluster**				
Other cluster (reference)	444	—	—	
Pheno5	57	0.57	0.28, 1.14	0.11
**ELN2017 risk**				
Intermediate (reference)	121	—	—	
Favorable	230	0.54	0.37, 0.80	<0.01
Adverse	150	1.23	0.85, 1.77	0.27
**Pheno5**				**0.030**
Pheno5 * Favorable		0.49	0.06, 3.98	
Pheno5 * Adverse		2.66	1.11, 6.41	

Only patients with complete data are shown.

Table S9. Multivariable Cox regression for event-free survival.

Characteristic	N	HR	95% CI	p-value
**Age**				
Age	501	1.00	0.99 – 1.02	0.5
**Disease type**				
De Novo (reference)	468	—	—	
Secondary	15	1.49	0.80 – 2.77	0.21
Treatment-related	18	1.05	0.56 – 1.99	0.87
**WBC**				
WBC	501	1.00	1.00, 1.00	0.6
**Patient cluster**				
Other cluster (reference)	444	—	—	
Pheno5	57	0.72	0.41 – 1.29	0.27
**ELN2017 risk**				
Intermediate (reference)	121	—	—	
Favorable	230	0.50	0.36 – 0.70	<0.001
Adverse	150	1.07	0.77 – 1.49	0.69
**Pheno5**				0.2
Pheno5 * Favorable		0.88	0.24 – 3.19	
Pheno5 * Adverse		1.96	0.90 – 4.25	

Only patients with complete data are shown.

Table S10. Genetic differences between ELN2017 intermediate risk patients with and without immunophenotypic patient cluster Pheno5.

Characteristic	Overall<br>N = 122	Intermediate w/ Pheno5<br>N = 25	Intermediate w/o Pheno5<br>N = 97	p-value
DNMT3A	40 (33%)	6 (24%)	34 (35%)	0.3
FLT3-ITD	37 (30%)	2 (8.0%)	35 (36%)	0.007
NPM1	27 (22%)	2 (8.0%)	25 (26%)	0.063
WT1	14 (11%)	0 (0%)	14 (14%)	0.071
NRAS	13 (11%)	1 (4.0%)	12 (12%)	0.3
IDH2^{R172}	13 (11%)	2 (8.0%)	11 (11%)	>0.9
IDH1	12 (9.8%)	2 (8.0%)	10 (10%)	>0.9
TET2	12 (9.8%)	2 (8.0%)	10 (10%)	>0.9
IDH2^{R140}	11 (9.0%)	0 (0%)	11 (11%)	0.12
FLT3-TKD	10 (8.2%)	0 (0%)	10 (10%)	0.2
PTPN11	10 (8.2%)	0 (0%)	10 (10%)	0.2
**t(11q;23)**	10 (8.2%)	1 (4.0%)	9 (9.3%)	0.7
**t(9;11)**	9 (7.4%)	1 (4.0%)	8 (8.2%)	0.7
BCOR	8 (6.6%)	2 (8.0%)	6 (6.2%)	0.7
SRSF2	8 (6.6%)	2 (8.0%)	6 (6.2%)	0.7
GATA2	7 (5.7%)	1 (4.0%)	6 (6.2%)	>0.9
EZH2	6 (4.9%)	0 (0%)	6 (6.2%)	0.3
STAG2	6 (4.9%)	3 (12%)	3 (3.1%)	0.10
KRAS	5 (4.1%)	0 (0%)	5 (5.2%)	0.6
**del(7q)**	5 (4.1%)	0 (0%)	5 (5.2%)	0.6

P-values calculated with Fisher's exact test. Only lesions with more than 5 occurrences are shown.

Table S11. Genetic differences between ELN2017 adverse risk patients with and without immunophenotypic patient cluster Pheno5.

Characteristic	Overall<br>N = 150	Adverse w/ Pheno5<br>N = 22	Adverse w/o Pheno5<br>N = 128	p-value
RUNX1	59 (39%)	6 (27%)	53 (41%)	0.2
Complex	48 (32%)	9 (41%)	39 (30%)	0.3
ASXL1	31 (21%)	6 (27%)	25 (20%)	0.4
FLT3-ITD	30 (20%)	0 (0%)	30 (23%)	**0.008**
DNMT3A	28 (19%)	3 (14%)	25 (20%)	0.8
TP53	28 (19%)	5 (23%)	23 (18%)	0.6
NRAS	25 (17%)	2 (9.1%)	23 (18%)	0.5
**del(5q)**	22 (15%)	4 (18%)	18 (14%)	0.7
SRSF2	21 (14%)	5 (23%)	16 (13%)	0.2
**Monosomy 17**	19 (13%)	3 (14%)	16 (13%)	>0.9
BCOR	17 (11%)	2 (9.1%)	15 (12%)	>0.9
**Monosomy 7**	17 (11%)	6 (27%)	11 (8.6%)	**0.021**
**Del(12p)**	16 (11%)	1 (4.5%)	15 (12%)	0.5
TET2	15 (10%)	0 (0%)	15 (12%)	0.13
IDH1	14 (9.3%)	3 (14%)	11 (8.6%)	0.4
SF3B1	13 (8.7%)	4 (18%)	9 (7.0%)	0.10
IDH2^{R140}	12 (8.0%)	3 (14%)	9 (7.0%)	0.4
**del(7q)**	12 (8.0%)	0 (0%)	12 (9.4%)	0.2
**del(17p)**	11 (7.3%)	2 (9.1%)	9 (7.0%)	0.7
PTPN11	10 (6.7%)	0 (0%)	10 (7.8%)	0.4
U2AF1	10 (6.7%)	0 (0%)	10 (7.8%)	0.4
WT1	10 (6.7%)	1 (4.5%)	9 (7.0%)	>0.9
**Monosomy 5**	10 (6.7%)	2 (9.1%)	8 (6.3%)	0.6

P-values calculated with Fisher's exact test. Only lesions with more than 10 occurrences are shown.

Table S12. Remission data for ELN2017 intermediate risk patients.

Group	w/o Pheno5	w/ Pheno5	p-value
**Remission timepoint**			0.36
Never in CR	9 (9.3%)	0 (0%)	
After cycle 1	57 (58.8%)	19 (76.0%)	
After cycle 2	25 (25.8%)	5 (20.0%)	
Later	6 (6.2%)	1 (4.0%)	

P-value calculated with Fisher's exact test.

Table S13. Remission data for ELN2017 adverse risk patients.

Group	w/o Pheno5	w/ Pheno5	p-value
**Remission timepoint**			0.85
Never in CR	15 (11.7%)	3 (13.5%)	
After cycle 1	65 (50.8%)	9 (40.9%)	
After cycle 2	34 (26.6%)	7 (31.8%)	
Later	14 (10.9%)	3 (13.6%)	

P-value calculated with Fisher's exact test.
